# Global genomic similarity and core genome sequence diversity of the *Streptococcus* genus as a toolkit to identify closely related bacterial species in complex environments

**DOI:** 10.7717/peerj.6233

**Published:** 2019-01-14

**Authors:** Hugo R. Barajas, Miguel F. Romero, Shamayim Martínez-Sánchez, Luis D. Alcaraz

**Affiliations:** 1 Departamento de Biología Celular, Facultad de Ciencias, Universidad Nacional Autónoma de México, Mexico City, Mexico; 2 Laboratorio Nacional de Ciencias de la Sostenibilidad, Instituto de Ecología. Universidad Nacional Autonóma de México, Mexico city, Mexico

**Keywords:** Genomic similarity score, Core genome, *Streptococcus*, Comparative genomics

## Abstract

**Background:**

The *Streptococcus* genus is relevant to both public health and food safety because of its ability to cause pathogenic infections. It is well-represented (>100 genomes) in publicly available databases. Streptococci are ubiquitous, with multiple sources of isolation, from human pathogens to dairy products. The *Streptococcus* genus has traditionally been classified by morphology, serum types, the 16S ribosomal RNA (rRNA) gene, and multi-locus sequence types subject to in-depth comparative genomic analysis.

**Methods:**

Core and pan-genomes described the genomic diversity of 108 strains belonging to 16 *Streptococcus* species. The core genome nucleotide diversity was calculated and compared to phylogenomic distances within the genus *Streptococcus*. The core genome was also used as a resource to recruit metagenomic fragment reads from streptococci dominated environments. A conventional 16S rRNA gene phylogeny reconstruction was used as a reference to compare the resulting dendrograms of average nucleotide identity (ANI) and genome similarity score (GSS) dendrograms.

**Results:**

The core genome, in this work, consists of 404 proteins that are shared by all 108 *Streptococcus*. The average identity of the pairwise compared core proteins decreases proportionally to GSS lower scores, across species. The GSS dendrogram recovers most of the clades in the 16S rRNA gene phylogeny while distinguishing between 16S polytomies (unresolved nodes). The GSS is a distance metric that can reflect evolutionary history comparing orthologous proteins. Additionally, GSS resulted in the most useful metric for genus and species comparisons, where ANI metrics failed due to false positives when comparing different species.

**Discussion:**

Understanding of genomic variability and species relatedness is the goal of tools like GSS, which makes use of the maximum pairwise shared orthologous sequences for its calculation. It allows for long evolutionary distances (above species) to be included because of the use of amino acid alignment scores, rather than nucleotides, and normalizing by positive matches. Newly sequenced species and strains could be easily placed into GSS dendrograms to infer overall genomic relatedness. The GSS is not restricted to ubiquitous conservancy of gene features; thus, it reflects the mosaic-structure and dynamism of gene acquisition and loss in bacterial genomes.

## Introduction

*Streptococcus* is a bacterial genus with more than 50 species. The health and environmental importance of streptococci include a diverse range of human and animal pathogens like the etiological agents for caries and meningitis as well as commensal species inhabiting the intestinal and respiratory tracts of animals (Kilian, 2007). Classification within the *Streptococcus* has been done all using the microbiological methods: morphology, biochemical tests, immunological tests, comparison of 16S ribosomal RNA (rRNA) gene phylogenies (Kawamura et al., 1995), and clinically using multi-locus sequence types (MLST) (Kawamura et al., 1995). Streptococci are divided into six main paraphyletic groups because of clinical or practical ease: pyogenes, mitis, anginosus, salivarius, bovis, and mutans according to the representative species for each clade (Kilian et al., 2008). Most of the streptococci were originally isolated from animal sources like humans, bovine, swine, and some from dairy products. Isolation source and general features of the strains used in this work are available (Table S1).

The current standards for bacteria phylogenetics, with genome sequences, are based on genome-wide average nucleotide identity (ANI) above 95% for estimating an overall genome-related index (Konstantinidis & Tiedje, 2005; Konstantinidis, Ramette & Tiedje, 2006; Chun et al., 2018). Traditionally, bacterial molecular phylogenetics rely upon 16S rRNA gene comparison with a 97% sequence identity cut-off to identify a bacterium species (Stackebrandt & Goebel, 1994). Protein translation is universal to cellular life, and thus the conservation of the molecular-associated machinery has been used as a molecular taxonomic marker due to its high conservation across the tree of life, including the 16S rRNA gene. However, 16S rRNA has a slow evolutionary rate which does not allow enough resolution to distinguish between closely related species (Stackebrandt & Goebel, 1994). The use of MLST is a standard practice for distinguishing between strains of pathogenic bacteria. Even what should define a bacterial species based upon its molecular phylogenetics is not well defined (Fraser et al., 2009).

The astounding amount of sequenced bacterial genomes (175,525 genome shotguns, 23,094 complete genomes in GenBank, November 2018) allows for pan-genomic phylogenomics (Tettelin et al., 2005; Liolios et al., 2010). The core genome for a set of related genomes is a concept that involves the identification of orthologous genes common to a species (Goodall et al., 2018) or genus (Alcaraz et al., 2010). The core genome should be discussed and analyzed yet its biological relevance tends to decrease if more genomes are added to the comparison. However, it does provide a set of genes that are probably responsible for a genus evolutionary cohesion. For example, 20 strains encompassing 13 species of the *Bacillus* genus were determined to share 814 core genes which defined specific genus features like the ability to build endospores (Alcaraz et al., 2010).

The core genome is automatically computable by software pipelines that identify shared orthologous genes (Contreras-Moreira & Vinuesa, 2013). Traditional phylogenetic reconstructions only use vertically inherited core genes while ignoring clade-specific genes. However, ignoring these clade-specific genes discards relevant elements of the biology of these organisms like horizontal gene transfer (HGT), gene family expansions, and gene content variability. Innocuous and pathogenic strains can be indistinguishable when using traditional phylogenetic methods. In this work, we used core genomes for each species to discriminate between species Streptococci-dominated metagenomes like the human mouth, where *Streptococcus* are differential for causing caries (*S. mutans*) or health (*S. dentisani*) *(*Belda-Ferre et al., 2012; Alcaraz et al., 2012; Camelo-Castillo et al., 2014; López-López et al., 2017). The reciprocal best hits (RBHs) in BLAST have been used to identify orthologs when comparing complete genomes (Moreno-Hagelsieb & Janga, 2007). The pairwise genome similarity score (GSS) values can define a distance matrix between a set of genomes, which can be turned into a distance dendrogram. Outgroups can be included in the comparison to place the root of the dendrogram. GSS ranges in values from 0 to 1. For example, when all orthologous proteins between two proteomes are identical, it has a maximum value of 1. Conversely, when two genomes have no similarity in orthologous protein, it has a value of 0 (Moreno-Hagelsieb & Janga, 2007).

Genome similarity score is a useful index to describe evolutionary distances between genomes using pairwise metrics that depend on normalized bit-score alignments of their predicted orthologs proteins (Janga & Moreno-Hagelsieb, 2004; Moreno-Hagelsieb & Janga, 2007; Moreno-Hagelsieb & Latimer, 2008; Alcaraz et al., 2010; Moreno-Hagelsieb et al., 2013). We think that GSS is a metric representing genomic distances from pairwise shared homologous genes is valuable to describe how related the strain to their relatives. In this work, we used core genomes for each species to discriminate between species Streptococci-dominated metagenomes like the human mouth, where *Streptococcus* are differential for causing caries (*S. mutans*) or health (*S. dentisani*) *(*Belda-Ferre et al., 2012; Alcaraz et al., 2012; Camelo-Castillo et al., 2014; López-López et al., 2017). We calculated the GSS score and generated a dendrogram for the 108 strains comprising 16 species of *Streptococcus.* The GSS results were then compared to 16S rRNA gene phylogeny and ANI dendrogram.

## Methods

### Analyzed genomes

Predicted proteomes for 108 strains of *Streptococcus*, representing 16 different species were downloaded from National Center for Biotechnology Information (NCBI) Genbank, based on a manually curated genome list (Table S1). The selected genomes list was curated previously to NCBI’s release of representative genomes update.

### Genomic similarity score

Orthologs were defined as RBH of pairwise comparisons using BLASTp (Camacho et al., 2009), with the following parameters: e-value = 1e^−6^, soft-masking, and Smith–Waterman algorithm to calculate bit scores. A minimum of 60% of the query length coverage was required. Detailed bioinformatic protocols are available as Data S1. Values of GSS have a range from 0 to 1 with a maximum reached when compared proteomes are identical. GSS is calculated as:

$${\rm{GSS}} = {{\sum\nolimits_{(i = 1)}^n {{\rm{compScor}}{{\rm{e}}_i}} } \over {\sum\nolimits_{(i = 1)}^n {{\rm{selfScor}}{{\rm{e}}_i}} }}.$$

The compScore is the sum of all the RBH BLAST bit scores of pairwise shared ortholog proteins divided by the sum of all RBH bit scores of the comparison with the reference proteomes themselves (self-Score). In the GSS, gene content is considered and nonmatching RBH adds a 0 value in the numerator. Since self-Score might differ in proteome *a* and *b*, the final GSS for the proteome pair *ab* is the arithmetic mean of GSS_*a*_ and GSS_*b*_. We used two bacilli species (*Bacillus subtilis* 168, and *B. licheniformis*) as out-groups for the comparisons of Streptococci GSS values. An inverse (1-GSS) distance matrix was built and used to compute a neighbor-joining tree using the APE library v. 3.5 (Paradis, Claude & Strimmer, 2004) for R v. 3.3.1 (R Core Team, 2018). GSS calculation scripts are available (Data S1).

A reference Streptococci phylogeny was built using 16S rRNA full-length sequence from each of the 108 streptococci genomes. Clustering of all the 16S rRNA gene copies (i. e., 4–7 copies in the analyzed genomes) within each genome was performed using *cd-hit-est* (Huang et al., 2010) clustering (97% identity) and in all genomes a single cluster was recovered. A representative, single copy 16S rRNA from each genome was used as input for multiple sequence alignment using structural models with SSU-ALIGN v. 0.1 (Nawrocki, 2009). The resulting 16S rRNA phylogeny was plotted using the neighbor-joining method from MEGA 5.2 (Tamura et al., 2013).

### Core genome calculations

Orthologs were defined as RBH of pairwise comparisons using the BLASTp program (Camacho et al., 2009), the following parameters were used as previously suggested (Moreno-Hagelsieb & Latimer, 2008). An E-value cutoff set to 1e^−6^, mask low complexity regions of the query sequence only during the search phase and perform an alignment with the Smith–Waterman algorithm to compute the bit score. Hits with an alignment length shorter than 60% of the length of the query sequence were discarded. Detailed scripting procedure of RBH is available (Data S1). The intersection set of all shared proteins of the 108 genomes constitute the RBH core genome. Only one protein per proteome is allowed per cluster using this methodology. From the RBH comparisons, pairwise global alignments were calculated to estimate the variance of sequence identity for each core protein using the Needleman–Wunsch method implemented in *needleall* of EMBOSS suite v. 6.6.0.0 (Rice, Longden & Bleasby, 2000).

Additionally, the core genome was also calculated using the software package GET_HOMOLOGUES (Contreras-Moreira & Vinuesa, 2013) with the BLASTp program to perform comparisons and the BDBH algorithm to define orthologous clusters. The minimum alignment coverage was set to 60% and the maximum E-value to 1e^−6^. Only clusters that included at least one sequence from all the analyzed genomes were considered for further analysis. Only protein coding genes were considered.

### Pan-genome calculation

The *Streptococcus* genus pan-genome was calculated by clustering all the predicted proteomes using cd-hit (Huang et al., 2010) with an identity cut-off value of 70%. This clustering method allows generating protein families without constraints of in-paralog groupings that collapses large gene families (i.e., ABC transporters). Additionally, GET_HOMOLOGUES was used as a second method to obtain the genus pan-genome. BLASTp (Camacho et al., 2009) hits with at least 70% sequence identity, a minimum of 75% alignment length coverage, and an E-value of 1e^−6^ were considered. The OrthoMCL algorithm (Li, Stoeckert & Roos, 2003) was used to group sequences. Only protein coding genes were considered.

### ANI calculation

Average nucleotide identity was calculated using *pyani* (Pritchard et al., 2016) for the 108 genomes used in this study (Table S1) with two methods: MUMmer v. 3.1 (Marçais et al., 2018) using minimum lengths of exact match (20 nt), maximum gaps (90 nt), and BLASTN+ (Camacho et al., 2009) with 1,020 nt windows.

### Core genome and pan-genome annotation

The core and pan-genomes were annotated using MG-RAST (Huang et al., 2010; Meyer et al., 2008) and their M5NR database (Wilke et al., 2012). Annotation required a minimum alignment length of 15 amino acids and 60% identity. Streptococci coding genes were uploaded to MG-RAST because it is possible to compare them with human oral metagenomes where *Streptococcus* species composition has repercussions for health or disease status (Belda-Ferre et al., 2012; Alcaraz et al., 2012; López-López et al., 2017).

### Metagenomic comparisons

Fragment recruitment analysis (Rusch et al., 2007) was done to compare oral metagenomes from healthy and diseased individuals against the *Streptococcus* reference core genome for each streptococci species using Nucmer from the MUMmer v. 3.1 (Marçais et al., 2018). A cut-off value of 90% identity (nucleotide) was chosen for classifying each metagenomic read to an individual species. Using minimum lengths of exact match (20 nt) and maximum gaps (90 nt).

## Results

### Core genome sequence diversity

According to the RBH method, the 108 streptococci compared share a core genome of just 404 proteins, which is a reduced number compared to the average protein content of 1,929 per strain. Both RBH and GET_HOMOLOGUES agreed in 255 core genes; only 51 detected by GET_HOMOLOGUES and 149 detected by RBH. The RBH strategy allows the detection of orthologous genes, meaning that in a pairwise comparison of genomes, for each sequence in genome A there can only be a unique sequence in genome B as the best match. Hence, this methodology does not allow in-paralogs. We allowed GET_HOMOLOGUES to use in-paralogs and the reported clusters were the ones shared the same sequence in all the compared genomes. The total pan-genome (cd-hit calculated) comprised 33,039 protein clusters (families) at 70% identity (Fig. S1). According to the GET_HOMOLOGUES data, the core genome is composed of 306 protein clusters and the pan-genome of 36,387 proteins.

**Figure 1:**
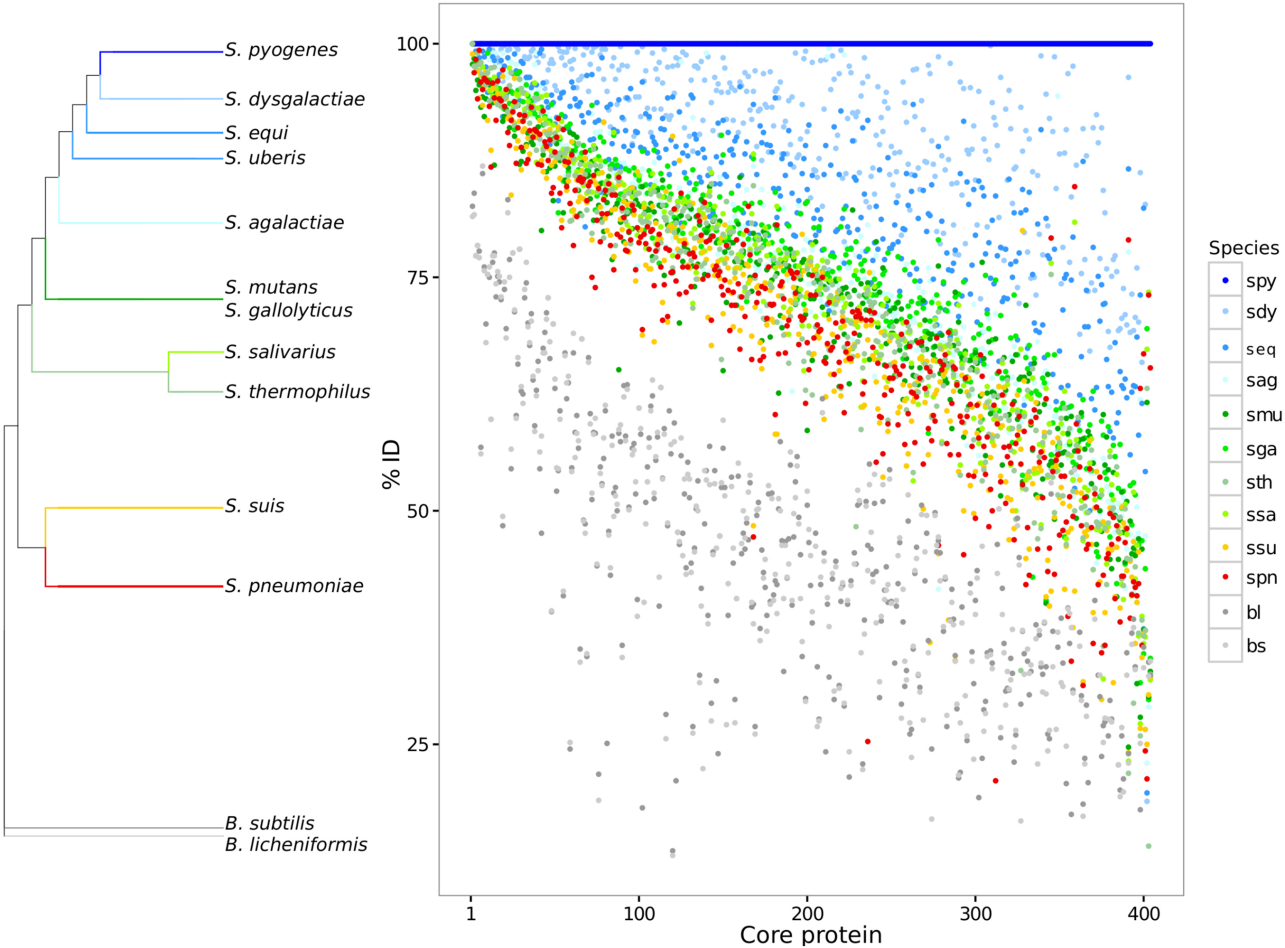
Core genome variability amongst different streptococci clades. Each core protein, for each streptococci species, was aligned against the reference *S. pyogenes.* The pairwise identity of each core protein, calculated by global sequence alignment, was sorted and plotted. The dendrogram shows a summary of genomic similarity score (GSS) distances. The identity variability highlights the species diversity even for the conserved coding genes. *S. pyogenes* (spy), *S. dysgalactiae* (sdy), *S. agalactiae*(sag), *S. parauberis* (spu), *S. iniae* (sin), *S.uberis* (sub), *S. equi* subsp. zooepidemicus (seq_z), *S. equi* ssp., equi (seq_z), *S. suis* (ssu), *S. thermophilus* (sth), *S. salivarius* (ssa), *S. mutans* (smu), *S. intermedius* (sint), *S. oligofermentans* (sol), *S. sanguinis* (ssan), *S. gordonii* (sgo), *S. parasanguinis* (sps), *S. pasteurianus* (spas), *S. oralis* (sor), *S. pneumoniae* (spn), *S. pseudopneumoniae* (sppn), *S. mitis* (smi), *S. gallolyticus* (sga), *S. macedonicus* (sma), *S. lutetiensis* (slu), *S. infantarius* (sinf), *B. subtilis* (bs), and *B. licheniformis* (bl).

Paired global alignments were performed with each core protein to analyze variation across species and strains over the core proteome. Each core protein cluster was plotted giving its pairwise sequence alignment identity to a reference sequence from *S. pyogenes* (Fig. 1). The species *S. pyogenes* was chosen as the reference because of its top phylogenetic position both in 16S and in GSS dendrogram (Fig. 1; Table S2). The high sequence identity (mean = 77.6 ± 11.5) for the core proteome suggests evidence for selective constraints. The range of protein sequence diversity in the core proteome ranges from 25% to 100% identity. Identity over the core genome is dependent on the evolutionary distance to the chosen reference. Based on the core proteome sequence diversity, we were able to describe a set of phylogenetic markers that can be used as DNA references to identify and discriminate between closely related species in metagenomes using high nucleotide identity cut-offs (>90%). Core genes for each of the streptococci species described here are available for the community in FASTA format (Data S2).

### Core genome functional analysis

Normalized abundances (*Z-scores*) of the pan-genome against the core were compared to the over-represented protein categories in the core (Fig. S2). The most abundant genes in the 404 protein core clusters found are related to translational machinery, including ribosomal proteins and translation-related proteins (Z = 3.08 core; Z = 0.88 pan-genome). There are more cell division related proteins in the core genome (Z = −0.87), than in the pan-genome (Z = −1.06). Membrane and cell envelope coding genes (M) are better represented in the core genome (Z = 0.22 and Z = 0.10 pan-genome, respectively). The most conserved core proteins (average pairwise identity >90%) are mostly related to the translation process and the 10 most matching are exclusively ribosomal proteins (Table S2). As average pairwise identity decreases for the core proteins, the appearance of several transport proteins, multiple transport-related proteins, transcriptional regulators, phosphatases, recombinases, peptidases, multidrug and efflux transporters (MATE), and hypothetical proteins occur (Table S2; Data S2). There are also a high proportion of core proteins present of unknown function (48 out of 404; 11.81%).

### Using the core genome to scan oral metagenomes

Metagenomic shotgun reads from oral microbiome samples were mapped to the core genomes to estimate relative abundance for each *Streptococcus* species. Oral metagenomes were chosen because of the presence of many streptococci in high abundance (4 to >20%) (Fig. S3). Two oral metagenomes were chosen: a patient with active caries and a healthy adult without caries (Belda-Ferre et al., 2012). In both metagenomes, the species with the most recruited number of fragments was *S. pneumoniae* (Fig. S4), but the caries etiological agent *S. mutans* was depleted (17 metagenomic fragments) in the caries-free individual (NOCA_01) and abundant (127 metagenomic fragments) in the patient with caries. Recruiting metagenomic sequences against each reference core genome and filtering alignments with high identity levels (≥90%) show that is possible to generate species-specific profiles (Fig. S4).

### Phylogenetic and genome similarity of the *Streptococcus* genus

**Figure 2:**
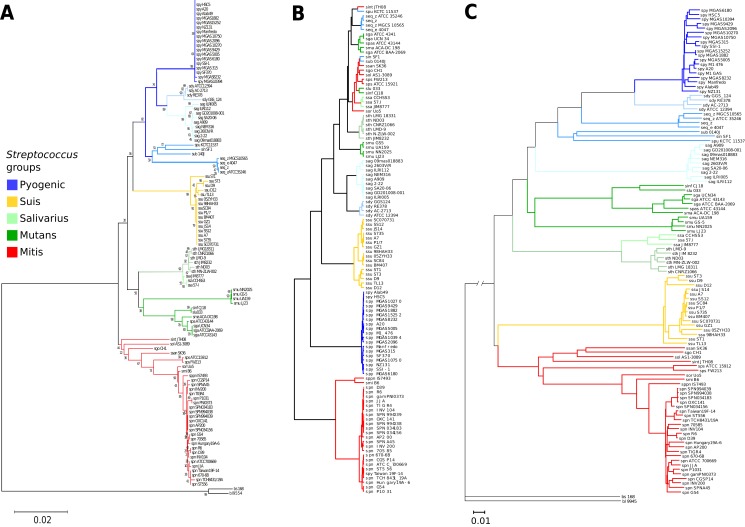
Genomic similarity score outperforms 16S rRNA strain resolution and solves genus-wide comparisons when compared to ANI. (A) Neighbor-joining 16S rRNA reconstruction, with 1,000 bootstraps. (B) Average nucleotide identity dendrogram. (C) Genomic similarity score (GSS) dendrogram. Some of the paraphyletic groups of streptococci are classified because clinical or practical uses (Kilian, 2007) are pyogenic, suis, salivarius, mutans, and mitis. The suis clade is rearranged closer to the mitis group, and resolution at the species level is achieved in the GSS dendrogram compared to single marker gene and ANI dendrograms. *S. pyogenes* (spy), *S. dysgalactiae* (sdy), *S. agalactiae*(sag), *S. parauberis* (spu), *S. iniae* (sin), *S.uberis* (sub), *S. equi* subsp. zooepidemicus (seq_z), *S. equi* ssp., equi (seq_z), *S. suis* (ssu), *S. thermophilus* (sth), *S. salivarius* (ssa), *S. mutans* (smu), *S. intermedius* (sint), *S. oligofermentans* (sol), *S. sanguinis* (ssan), *S. gordonii* (sgo), *S. parasanguinis* (sps), *S. pasteurianus* (spas), *S. oralis* (sor), *S. pneumoniae* (spn), *S. pseudopneumoniae* (sppn), *S. mitis* (smi), *S. gallolyticus* (sga), *S. macedonicus* (sma), *S. lutetiensis* (slu), *S. infantarius* (sinf), *B. subtilis* (bs), and *B. licheniformis* (bl).

A 16S rRNA phylogenetic reconstruction was done as a reference and confirms previously proposed clades (Fig. 2A) (Kawamura et al., 1995). There is a pyogenic clade containing multiple species: *S. pyogenes*, *S. dysgalactiae*, *S. equi*, *S. uberis*, *S. parauberis*, *S. agalactiae*, and *S. pneumoniae.* A second clade is the salivarius group formed just by *S. thermophilus* and *S. salivarius.* The mutans clade groups the following species: *S. mutans*, *S. infantarius*, *S. lutetiensis*, *S. macedonicus*, and *S. gallolyticus.* The *S. suis* has its clade with multiple strains of the same species. A fifth clade known as the mitis group is the basal group: *S. pneumoniae*, *S. pseudopneumoniae*, *S. mitis*, *S. pasteurianus*, *S. parasanguinis*, *S. sanguinis*, *S.gordonii*, *S. oligofermentans*, and *S. intermedius*. The external groups are *B. subtilis* and *B. licheniformis*.

Average nucleotide identity was calculated for all the Streptococci genomes and it was able to discriminate between main pyogenic and suis clades (Fig. 2B). However, ANI did not differentiate the mutans and salivarius groups, which are supported both by 16S phylogeny and GSS dendrogram (Figs. 2A and 2C, respectively). Interestingly, there is an ANI clade formed by a mix of pyogenic, mitis, and salivarius groups, not supported by either GSS or 16S phylogeny. The mixed streptococci group was analyzed, and we found that the clustering is due to false positives that have a low number of nucleotide regions aligned with high identity which distort the ANI result. A graphical example of the false positives was calculated showing the overall genomic coverage between neighboring strains misplaced by ANI (Fig. S5). The ANI is based in average values of identified homologous genomic regions, without length or number of alignments correction (Fig. S5; Table S3), thus resulting in incorrect clusters. We then calculated the ANI using the same neighbor-joining algorithms with the raw ANI values (Table S3) to calculate branch distances and the ANI values did not cluster in the same groups described by 16S rRNA gene nor GSS (Fig. S6). The complete ANI correlogram is available (Fig. S7).

The GSS dendrogram has the same clades as the 16S rRNA (Fig. 2C), however, GSS rearranged the pyogenic group, where *S. agalactiae* is included interior to the pyogenic clade in the 16S phylogeny and GSS shows *S. agalactiae* as the basal group for the pyogenic clade. Another rearrangement of GSS is the suis group, which is normally a sister clade to the mitis group, but in the 16S rRNA phylogeny, suis is placed as a sister clade to the pyogenic group. It is noticeable that the GSS dendrogram distances are vast enough to distinguish discrete groups among closely related strains like such as the inner clades of suis, pyogenic, mutans, and mitis groups. There are resolved clades in the GSS dendrogram for strains of *S. pneumoniae* and *S. pseudopneumoniae*; whereas, 16S rRNA does not distinguish close relationships, but instead allows polytomies. Also, the suis GSS clade shows resolved branching when comparing to the 16S rRNA phylogeny.

## Discussion

*Streptococcus* species have historically been classified by their cell wall antigenic properties (Kayser, Bienz & Eckert, 2011) and the clinical criteria for pathogenic strains (e.g., hemolysis capability). More recently, molecular phylogenetics has aided in classification of streptococci (Kawamura et al., 1995; Kilian et al., 2008). Analysis of genomic variability within the same species expanded with the definition of relevant concepts like the pan-genome and the core genome for *S. agalactiae* (Tettelin et al., 2005).

The core genome is dependent on the set of genomes being analyzed, for each genome added, the size of the core would decrease if any genes are not present for that genome. Additionally, different methods can also estimate diverse core and pan-genome sizes as shown in previous studies (Fouts et al., 2012). In this work, 404 core proteins comprised the core genome according to the RBH method in the 108 strains compared, while GET_HOMOLOGUES calculate 306 proteins. Historically, the first core genome for streptococci was 611 genes for 26 genomes (Lefébure & Stanhope, 2007), the second effort wast 547 genes for 64 genomes (Van den Bogert et al., 2013), and a third reconstruction gave 369 core genes for 138 strains (Gao et al., 2014). Of note, 11.81% of the core genes of streptococci are of unknown function (Table S2; Data S2) and may represent an opportunity for therapeutic targeting. The core genome of bacteria, no matter the species, genus or analysis method should be an open repository, recalculated each time a new strain is sequenced, and then shared with the scientific community. This raises the possibility of the creation of a database that self-updates with new genome discoveries. The core genome for streptococci provides a platform for investigating what is essential to the lifestyle of these organisms and can be used to analyze their presence in metagenomic samples. Additionally, we think that traditional phylogenetic methodology is necessary to understand vertical group evolution and GSS, or similar measures of whole genome relatedness, are an improvement over marker gene-based methods. However, bacteria have amazing capabilities to transfer genes by conjugation, transformation, and competence, with high rates of recombination that pose a challenge for traditional phylogenetics (Frost et al., 2005; Francino, 2012). The pan-genomic analysis shows the variability within a species, which may indicate adaptation to specific environments by additions or deletions to the genomic repertoire (Tettelin et al., 2008; Mira et al., 2010; Vernikos et al., 2015). The GSS measures bacterial strain similarity over all homologous genetic elements shared by a pair of bacteria, no matter if it is vertically or horizontally transmitted, including the entire pan-genome within its calculation (Janga & Moreno-Hagelsieb, 2004; Moreno-Hagelsieb & Janga, 2007; Alcaraz et al., 2010; Moreno-Hagelsieb et al., 2013). New standards in expanding the bacterial taxonomic rules by making use of whole genome information is being established and ANI is the preferred choice to discriminate between species (Chun et al., 2018). Working within the genus-level involves methods that are able to identify homologous sequences; here we found protein sequence diversity with distances spanning from 100% to less than 25% identity for the global alignments. The main advantage of GSS is that it uses both core and pan-genomic information to estimate relatedness between strains. Proteins are the preferred choice to find homologs with large evolutionary distances (Rost, 1999). The ANI analysis method is preferred when comparing within strains of the same species (Chun et al., 2018), but it discards homologous information. The shortcoming of comparing nucleotides in long time diverging lineages with ANI are evident as there are estimates that the last common ancestor of Streptococci occurred about 0.5 billion years ago (Battistuzzi, Feijao & Hedges, 2004). Multiple sequenced redundancy in strains complicates comparative genome analysis as information beyond nucleotide clustering is needed. Eliminating genome redundancy with information like distance matrix or phylogenetic information by using GGRaSP (Clarke et al., 2018) combined with the GSS approach could easily integrate to both methodologies may provide for a superior analysis.

The GSS dendrogram is consistent with the accepted clades of streptococci. GSS provides better resolution of clade structure and distances than the 16S rRNA gene-based phylogeny (Fig. 2). Within-group resolution is much improved in the GSS dendrogram for several streptococci species like *S. pyogenes*, *S. suis*, *S. mutans*, and *S. pneumoniae*, which are practically indistinguishable using 16S, but GSS shows monophyletic clades for each species with explicit branching and long enough distances to identify each strain within a species (Fig. 2C).

The growth of metagenomic data needs a framework to distinguish between closely related strains. Some environments host intra-genus diversity with implications for health such as human vaginal microbiomes dominated by *Lactobacillus* species (Gajer et al., 2012) and human oral microbiome (Belda-Ferre et al., 2012; Simón-Soro et al., 2013). There are multiple ways to bin metagenomic diversity; from nucleotide *k-mer* frequencies (Ulyantsev et al., 2016), using phylogenomic markers (Segata et al., 2012), AMPHORA (Segata et al., 2012; Kerepesi, Bánky & Grolmusz, 2014), by annotation of ribosomal genes (Pruesse et al., 2007; Cardenas et al., 2009), and lowest common ancestor binning (Huson et al., 2007; Meyer et al., 2008). In this work, the use of the core genome of a genus provides a relatively simple (404 genes) dataset to align and recruit metagenomic information (e.g., reads, contigs) to estimate species abundances based on the coverage and identity of each aligned fragment (Fig. S4). Despite the biological relevance, or connecting it to essential genes (Goodall et al., 2018), the core genome of a specific clade provides a resource to discriminate between closely related strains. Sequence identity variation within the core genome provides a basis for understanding the differential selective pressure for each core cluster (Table S2). Core genome variation could be a significant input for practical clinical applications like probe and diagnosis designs or to choose therapeutic targets using conserved but highly variable proteins.

The methods used to calculate ANI are based on alignable regions between pairs of compared species from complete contigs resulting from shotgun genome sequencing assemblies to completed sequenced and assembled genomes. The ANI is calculated as the ANI within matching regions likewise hyper-conserved genes (rRNA) and promiscuous genomic regions resulting from HGT (Pritchard et al., 2016). If there are few alignable genomic regions but with high identity, false positives are expected. ANI is a reliable and fast tool to compare strains within the same bacterial species and match to newly sequenced ones.

A long-range genomic comparison, like the genus-wide presented here, amino acids are a far superior choice for deep-diverging organisms like bacteria. The amino acid search of homologous proteins reduces the number of false positives, increases the chance of finding remote homologous sequences, and eliminates missed alignments because of differential codon usage in each species. Sequence conservation in amino acids, due to functional constraints in the proteins, reduces the amount of sequence while adding the possibility of similar changes. The GSS takes all the perks of using amino acids for homologous searching when compared to ANI or individual genes used as phylogenetic markers. Additionally, the GSS offers a real-world advantage for most current bacterial draft genome sequences; it can identify most of the pairwise shared orthologs to estimate a global similarity using bit scores that summarize alignment quality. Finally, the alignment quality is a proxy to the evolutionary story of the compared organisms. The previously exposed reasons support the GSS a reliable option to perform phylogenomic analysis and place newly sequenced bacteria in context while including the genomic mosaicism of bacteria which is closer to their genome dynamics that only account for universal shared genes to make phylogenetic inferences.

## Conclusions

In this work, we present a comparative genomics analysis using the streptococci core genome with a proposal of 404 conserved proteins in 108 strains belonging to 16 *Streptococcus* species. The core genome represents the phylogenetic coherence of the group and each protein can be used as a phylogenetic marker. In addition to their functionality as phylogenetic markers, core genes allow making a metabolic inventory of what proteome functions are essential for this genus. In the streptococci core genome, the most common phylogenetic markers such as ribosomal biogenesis genes are expectedly reported, but new therapeutic targets arise with genes such as phosphatases, transporters, and even hypothetical conserved proteins. The utility of GSS to maximize the amount of comparative homologous information is demonstrated, being practical to resolve genomic similarity relationships among genomes using the maximum amount of genomic information shared by pairs of comparison genomes. The GSS has the resolution to distinguish within similar strains while avoiding false positives as those observed with the ANI in genus-level comparisons. Finally, the GSS can be used as a proxy for the genomic dynamics and evolutionary history of microorganisms.

## Supplemental Information

10.7717/peerj.6233/supp-1 Supplemental Information 1 Genome sequences and their environmental sources used in this work. Information included: Strains names, CDS number, NCBI accession numbers, isolation sources.

10.7717/peerj.6233/supp-2 Supplemental Information 2 Global average identity scores, annotation, sequence identifiers for the core proteome of the streptococci.

10.7717/peerj.6233/supp-3 Supplemental Information 3 Raw ANI values matrix for the streptococci.

10.7717/peerj.6233/supp-4 Supplemental Information 4 Detailed bioinformatic protocols. GSS calculations jupyter notebook.

10.7717/peerj.6233/supp-5 Supplemental Information 5 ZIP file containing FASTA files for each streptococci species core genome.

10.7717/peerj.6233/supp-6 Supplemental Information 6 Core genome and pan-genome plots for the 108 streptococci strains. Additionally, each streptococci species core genome and orthologous genes shared between strains.

10.7717/peerj.6233/supp-7 Supplemental Information 7 *Streptococcus* core and pan-genome summary of general functions profiles according to the Cluster of Orthologous Groups (COGs).

10.7717/peerj.6233/supp-8 Supplemental Information 8 Metagenomic abundances of Streptococcus in metagenomic samples. Calculated by core genome fragment recruitment, the lowest common ancestor (LCA), and 16S rRNA gene abundances.

10.7717/peerj.6233/supp-9 Supplemental Information 9 Metagenomic fragment recruitment against Streptococci core genomes. (A) Histogram showing the species gene relative abundance in metagenomic reads from a caries patient. (B) Metagenomic reads from a patient with caries, and C) Metagenomic reads from a healthy individual (caries-free) were aligned against the core genomes of 10 different species of Streptococci. (D) Histogram representing the species gene relative abundance in metagenomic reads from a healthy individual. Species-specific profiles can be generated by this method. *S. mutans* is depleted in the healthy individual while abundant metagenomic reads are identified in caries patient.

10.7717/peerj.6233/supp-10 Supplemental Information 10 Mummer alignments of ANI false-positive clustering along with the ANI values, each strain misallocation is showed in the ANI dendrogram.

10.7717/peerj.6233/supp-11 Supplemental Information 11 ANI dendrogram using Neighbor-Joining along 16S rRNA gene phylogeny and GSS dendrogram.

10.7717/peerj.6233/supp-12 Supplemental Information 12 ANI correlogram for the selected 108 streptococci strains.

## Additional Information and Declarations

### Data Availability

The following information was supplied regarding data availability: The raw data are provided in the Supplemental Files.
