# Supplementary material for: Global genomic similarity and core genome sequence diversity of the Streptococcus genus as a toolkit to identify closely related bacterial species in complex environments
Source: PeerJ. 2019 Jan 14;6:e6233. doi: 10.7717/peerj.6233 (PMC6336011; doi:10.7717/peerj.6233)
Supplement: Supplemental Information 1 — Information included: Strains names, CDS number, NCBI accession numbers, isolation sources. [file peerj-07-6233-s001.pdf]

Strain name	CDS	Accession	Isolation source	Lifestyle	Reference
Streptococcus pyogenes SF370	1696	NC_002737	*Homo sapiens: infected wound*	Pathogen	Ferreti et al., 2001
Streptococcus pneumoniae TIGR4	2105	NC_003028	*Homo sapiens: blood culture*	Pathogen	Tettelin et al., 2001
Streptococcus pneumoniae R6	2042	NC_003098	*Homo sapiens*	Pathogen	Hoskins et al., 2001
Streptococcus pyogenes MGAS8232	1839	NC_003485	*Homo sapiens: throat, rheumatic fever*	Pathogen	Beres et al., 2002
Streptococcus pyogenes MGAS315	1865	NC_004070	*Homo sapiens: STSS*	Pathogen	Beres et al., 2002
Streptococcus agalactiae 2603V/R	2124	NC_004116	*Homo sapiens: clinical isolate*	Pathogen	Tettelin et al., 2001
Streptococcus mutans UA159	1960	NC_004350	*Homo sapiens: active dental caries*	Pathogen	Ajdić et al., 2002
Streptococcus agalactiae NEM316	2094	NC_004368	*Homo sapiens: fatal septicemia*	Pathogen	Glaser et al., 2002
Streptococcus pyogenes SSI-1	1859	NC_004606	*Homo sapiens: toxic shock*	Pathogen	Nakagawa et al., 2003
streptococcus pyogenes MGAS10394	1886	NC_006086	*Homo sapiens: pharynx*	Pathogen	Banks et al., 2004
Streptococcus thermophilus LMG 18311	1888	NC_006448	*Yoghurt in Francia*	Non Pathogen	Bolotin et al., 2004
Streptococcus thermophilus CNRZ1066	1915	NC_006449	*Yoghurt*	Non Pathogen	Bolotin et al., 2004
Streptococcus pyogenes MGAS6180	1894	NC_007296	*Homo sapiens*	Pathogen	Green et al., 2005
Streptococcus pyogenes MGAS5005	1865	NC_007297	*Homo sapiens*	Pathogen	Sumby et al., 2005
Streptococcus agalactiae A909	1996	NC_007432	*Homo sapiens: newborn in sepsis*	Pathogen	Tettelin et al., 2005
Streptococcus pyogenes MGAS9429	1877	NC_008021	*Homo sapiens: pharynx*	Pathogen	http://genomesonline.org/ cgi-bin/GOLD/GOLDCards.cgi? goldstamp=Gc00379
Streptococcus pyogenes MGAS10270	1987	NC_008022	*Homo sapiens: faringe*	Pathogen	http://genomesonline.org/ cgi-bin/GOLD/GOLDCards.cgi? goldstamp=Gc00378
Streptococcus pyogenes MGAS2096	1898	NC_008023	*Homo sapiens: poststreptococal Glomerulonephritis*	Pathogen	http://genomesonline.org/ cgi-bin/GOLD/GOLDCards.cgi? goldstamp=Gc00377
Streptococcus pyogenes MGAS10750	1978	NC_008024	*Homo sapiens: pharynx*	Pathogen	http://genomesonline.org/ cgi-bin/GOLD/GOLDCards.cgi? goldstamp=Gc00376
Streptococcus thermophilus LMD-9	1715	NC_008532, N NC_008501	*NA*	NA	Makarova et al., 2006
Streptococcus pneumoniae D39	1914	NC_008533	*Homo sapiens*	Pathogen	Lanie et al., 2007
Streptococcus sanguinis SK36	2270	NC_009009	*Homo sapiens: dental plaque*	Pathogen Oportunista	Xu et al., 2007
Streptococcus pyogenes Str. Manfredo	1745	NC_009332	*Homo sapiens: rheumatic fever*	Pathogen	Holden et al., 2007
Streptococcus suis 05ZYH33	2186	NC_009442	*Homo sapiens: STSS*	Pathogen	Chen et al., 2007
Streptococcus suis 98HAH33	2185	NC_009443	*Homo sapiens: STSS*	Pathogen	Chen et al., 2007
Streptococcus gordonii str. Challis substr. CH1	2051	NC_009785	*Homo sapiens*	Pathogen	Vickerman et al., 2007
Streptococcus pneumoniae Hungary19A-6	2155	NC_010380	*Homo sapiens: inner ear*	Pathogen	http://genomesonline.org/cgi-bin/GOLD/GOLDCards.cgi? goldstamp=Gc00735
Streptococcus pneumoniae CGSP14	2206	NC_010582	*Homo sapiens: necrotizing pneumonia*	Pathogen	Ding et al., 2009
Streptococcus pneumoniae G54	2114	NC_011072	*Homo sapiens: respiratory tract*	Pathogen	Dopazo et al., 2001
Streptococcus equi subsp. Zooepidemicus MGCS10565	1893	NC_011134	*Homo sapiens throat*	Pathogen	Beres et al., 2008
Streptococcus pyogenes NZ131	1700	NC_011375	*Homo sapiens: glomerulonephritis*	Pathogen	McShan et al., 2008
Streptococcus pneumoniae ATCC 700669	1990	NC_011900	*Homo sapiens*	Pathogen	Croucher et al., 2009
Streptococcus uberis 0140J	1762	NC_012004	*Bovine*	Pathogen	Ward et al., 2009
Streptococcus pneumoniae JJA	2123	NC_012466	*Homo sapiens*	Pathogen	http://genomesonline.org/ cgi-bin/GOLD/GOLDCards.cgi? Goldstamp=Gc00973
Streptococcus pneumoniae P1031	2073	NC_012467	*Homo sapiens*	Pathogen	http://genomesonline.org/ cgi-bin/GOLD/GOLDCards.cgi? goldstamp=Gc00972
Streptococcus pneumoniae 70585	2202	NC_012468	*Homo sapiens*	Pathogen	http://genomesonline.org/ Cgi-bin/GOLD/GOLDCards.cgi? Goldstamp=Gc00969
Streptococcus pneumoniae Taiwan19F-14	2044	NC_012469	*Homo sapiens: cerebrospinal fluid*	Pathogen	http://genomesonline.org/ cgi-bin/GOLD/GOLDCards.cgi? goldstamp=Gc00974
Streptococcus equi subsp. Zooepidemicus	1869	NC_012470	*Horse*	Pathogen	Holden et al., 2009
Streptococcus equi subsp. Equi 4047	2000	NC_012471	*Horse*	Pathogen	Holden et al., 2009
Streptococcus dysgalactiae Subsp. equisimilis GGS_124	2094	NC_012891	*Homo sapiens: STSS*	Pathogen	Shiomura et al., 2011

Strain name	CDS	Accession	Isolation source	Lifestyle	Reference
Streptococcus suis SC84	1898	NC_012924	*Homo sapiens: STSS-L*	Pathogen	Holden et al., 2009
Streptococcus suis P1/7	1824	NC_012925	*Sus scrofa & Homo sapiens*	Pathogen	Boyle et al., 2012
Streptococcus suis BM407	1947	NC_012926, N[illegible]	*Homo sapiens: meningitis*	Pathogen	Holden et al., 2009
Streptococcus gallolyticus UCN34	2223	NC_013798	*Homo sapiens*	Pathogen	Rusniok et al., 2010
Streptococcus mitis B6	2004	NC_013853	*Homo sapiens*	Pathogen	Denapaite et al., 2010
Streptococcus mutans NN2025	1895	NC_013928	*Homo sapiens: dental caries*	Pathogen	Maruyama et al., 2009
Streptococcus pneumoniae TCH8431/19A	2275	NC_014251	*Homo sapiens: respiratory tract*	Pathogen	http://genomesonline.org/cgi-bin/GOLD/GOLDCards.cgi?goldstamp=Gc01351
Streptococcus pneumoniae AP200	2216	NC_014494	*Homo sapiens: LCE: meningitis*	Pathogen	Camilli et al., 2008
Streptococcus pneumoniae 670-6B	2352	NC_014498	*Homo sapiens*	Pathogen	Donati et al., 2010
Streptococcus gallolyticus subsp. Gallolyticus ATCC BAA-2069	2329	NC_015215, N[illegible]	*Homo sapiens: blood culture*	Pathogen	Camilli et al., 2008
Streptococcus oralis Uo5	1907	NC_015291	*Homo sapiens: oral cavity*	Pathogen	Reichmann et al., 2011
Streptococcus suis ST3	1952	NC_015433	*Sus scrofa: pneumonia*	Pathogen	Hu et al., 2011
Streptococcus parauberis KCTC 11537	1868	NC_015558	*Diseased fish (Paralichthys sp.)*	Pathogen	Nho et al., 2011
Streptococcus pasteurianus ATCC 43144	1869	NC_015600	*Homo sapiens: blood culture*	Pathogen	Lin et al., 2011
Streptococcus parasanguinis ATCC 15912	2022	NC_015678	*Homo sapiens: garganta*	Pathogen	http://genomesonline.org/cgi-bin/GOLD/GOLDCards.cgi?Goldstamp=Gc01842
Streptococcus salivarius CCHSS3	2027	NC_015760	*Homo sapiens: blood culture*	Pathogen	Delorme et al., 2011
Streptococcus pseudopneumoniae IS7493	2236	NC_015875, N[illegible]	*Homo sapiens: sputum*	Pathogen	Shahinas et al., 2011
Streptococcus macedonicus ACA-DC 198	1994	NC_016749, N[illegible]	*Kasseri cheese*	Non Pathogen	Papadimitriou et al., 2012
Streptococcus infantarius Subsp. infantarius CJ18	1906	NC_016826, N[illegible]	*Camel milk*	Non Pathogen	Jans et al., 2012
Streptococcus pyogenes MGAS15252	1662	NC_017040	*Homo sapiens: white tissue infection*	Pathogen	Fittipaldi et al., 2012
Streptococcus pyogenes MGAS1882	1691	NC_017053	*Homo sapiens*	Pathogen	Fittipaldi et al., 2012
Streptococcus thermophilus ND03	1919	NC_017563	*Chinese dairy products*	Non Pathogen	Sun et al., 2011
Streptococcus dysgalactiae Subsp. equisimilis ATCC 12394	2056	NC_017567	*Bovine udder*	Pathogen	Zuzuki et al., 2011
Streptococcus gallolyticus subsp. Gallolyticus ATCC 43143	2246	NC_017576	*Homo sapiens: blood culture*	Pathogen	Lin et al., 2011
Streptococcus thermophilus JIM 8232	2145	NC_017581	*Milk*	Non Pathogen	Delorme et al., 2011
Streptococcus equi subsp. Zooepidemicus ATCC 35246	2087	NC_017582	*Sus scrofa*	Pathogen	Ma et al., 2011
Streptococcus pneumoniae INV104	1820	NC_017591	*Homo sapiens*	Pathogen	Donati et al., 2010
Streptococcus pneumoniae OXC141	1823	NC_017592	*Homo sapiens*	Pathogen	Donati et al., 2010
Streptococcus pneumoniae INV200	1929	NC_017593	*Homo sapiens*	Pathogen	Donati et al., 2010
Streptococcus salivarius 57.I	1941	NC_017594	*Homo sapiens*	Pathogen	Geng et al., 2011
Streptococcus salivarius JIM8777	1979	NC_017595	*Homo sapiens: healthy oral cavity*	Non Pathogen	Guédon et al., 2011
Streptococcus pyogenes Alab49	1773	NC_017596	*Homo sapiens: impetigo*	Pathogen	Bessen et al., 2011
Streptococcus suis GZ1	1977	NC_017617	*Homo sapiens*	Pathogen	Ye et al., 2009
Streptococcus suis JS14	2066	NC_017618	*Homo sapiens*	Pathogen	Holden et al., 2009
Streptococcus suis SS12	2079	NC_017619	*NA*	NA	Zhang et al., 2011
Streptococcus suis D9	2074	NC_017620	*NA*	Pathogen	Zhang et al., 2011
Streptococcus suis D12	2078	NC_017621	*NA*	Pathogen	Zhang et al., 2011
Streptococcus suis A7	1974	NC_017622	*Homo sapiens*	Pathogen	Zhang et al., 2011
Streptococcus mutans LJ23	1921	NC_017768	*Homo sapiens: oral cavity*	Pathogen	Aikawa et al., 2012
Streptococcus pneumoniae ST556	2148	NC_017769	*Homo sapiens*	Pathogen	Li et al., 2012
Streptococcus parasanguinis FW213	2019	NC_017905	*Homo sapiens: dental plaque*	Non Pathogen	Geng et al., 2012
Streptococcus thermophilus MN-ZLW-002	1910	NC_017927	*Chinese dairy products*	Non Pathogen	Kang et al., 2012
Streptococcus suis ST1	1987	NC_017950	*NA*	NA	Zhang et al., 2011
Streptococcus intermedius JTH08	1702	NC_018073	*Homo sapiens: hepatic abscess*	Pathogen	Tomoyasu et al., 2010
Streptococcus mutans GS-5	1878	NC_018089	*Homo sapiens: dental caries*	Pathogen	Biswas et al., 2012
Streptococcus suis S735	1882	NC_018526	*Sus scrofa*	Pathogen	Boyle et al., 2012
Streptococcus pneumoniae SPNA45	1926	NC_018594	*NA*	NA	Donati et al., 2010

Strain name	CDS	Accession	Isolation source	Lifestyle	Reference
Streptococcus pneumoniae GamPNI0373	2119	NC_018630	*Homo sapiens: nasopharynx*	Pathogen	http://genomesonline.org/cgi-bin/GOLD/GOLDCards.cgi?Goldstamp=Gc02735
Streptococcus agalactiae GD201008-001	1964	NC_018646	*Tilapia: meningoencephalitis*	Pathogen	Liu et al., 2012
Streptococcus dysgalactiae Subsp. equisimilis RE378	1877	NC_018712	*Homo sapiens: invasive infection*	Pathogen	Okumura et al., 2012
Streptococcus pyogenes A20	1828	NC_018936	*Homo sapiens: necrotizing fascitis*	Pathogen	Zheng et al., 2013
Streptococcus dysgalactiae Subsp. equisimilis AC-2713	2215	NC_019042	*Homo sapiens: blood culture*	Pathogen	Watanabe et al., 2013
Streptococcus agalactiae SA20-06	1710	NC_019048	*Tilapia*	Pathogen	Pereira et al., 2013
Streptococcus suis SC070731	1933	NC_020526	*NA*	NA	Wu et al., 2014
Streptococcus pyogenes M1 476	1572	NC_020540	*Homo sapiens: STSS*	Pathogen	Miyoshi-Akiyama et al., 2012
Streptococcus pneumoniae SPN994039	1819	NC_021005	*Homo sapiens*	Pathogen	Donati et al., 2010
Streptococcus pneumoniae SPN034156	1799	NC_021006	*Homo sapiens*	Pathogen	Donati et al., 2010
Streptococcus pneumoniae SPN994038	1819	NC_021026	*Homo sapiens*	Pathogen	Donati et al., 2010
Streptococcus pneumoniae SPN034183	1819	NC_021028	*Homo sapiens*	Pathogen	Donati et al., 2010
Streptococcus oligofermentans AS 1.3089	2069	NC_021175	*Homo sapiens: Healthy teeth*	Non Pathogen	Tong et al., 2013
Streptococcus agalactiae 2-22	1548	NC_021195	*Trouts in Israel*	Pathogen	Rosinski-Chupin et al., 2013
Streptococcus suis TL13	1939	NC_021213	*NA*	NA	Wang et al., 2013
Streptococcus iniae SF1	2125	NC_021314	*NA*	NA	NA
Streptococcus agalactiae 09mas018883	2089	NC_021485	*Milk from cow with clinical mastitis In Switzerland*	Pathogen	Zubair et al., 2013
Streptococcus agalactiae ILRI005	2155	NC_021486	*Camelus dormedarius*	Pathogen	Zubair et al., 2013
Streptococcus agalactiae ILRI112	2073	NC_021507	*Camelus dormedarius*	Pathogen	Zubair et al., 2013
Streptococcus pyogenes HSC5	1744	NC_021807	*NA*	Pathogen	Port et al., 2013
Streptococcus lutetiensis 033	1890	NC_021900	*Homo sapiens: feces*	Pathogen	Jin et al., 2013
Bacillus subtilis str. 168	4175	NC_000964	*NA*	NA	Barbe et a., 2009
Bacillus licheniformis 9945A	4225	NC_021362	*NA*	NA	Rachinger et al., 2013

References for Supplementary Table 1.

Aikawa C., Furukawa N., Watanabe T., Minegishi K., Furukawa A., Eishi Y., Oshima K., Kurokawa K., Hattori M., Nakano K., Maruyama F., Nakagawa I., Ooshima T. 2012. Complete genome sequence of the serotype k *Streptococcus mutans* strain LJ23. *Journal of bacteriology* 194:2754–5. DOI: 10.1128/JB.00350-12.

Ajdić D., McShan WM., McLaughlin RE., Savić G., Chang J., Carson MB., Primeaux C., Tian R., Kenton S., Jia H., Lin S., Qian Y., Li S., Zhu H., Najar F., Lai H., White J., Roe B a., Ferretti JJ. 2002. Genome sequence of *Streptococcus mutans* UA159, a cariogenic dental pathogen. *Proceedings of the National Academy of Sciences of the United States of America* 99:14434–9. DOI: 10.1073/pnas.172501299.

Banks D., Porcella S., Barbian K., Beres S., Philips L., Voyich J., DeLeo F., Martin J. 2004. Progress toward Characterization of the Group A S*treptococcus* Metagenome: Complete Genome Sequence of a Macrolide-Resistnat Serotype M6 Strain. *The Journal of Infectious Disease* 190:727–782.

Barbe V., Cruveiller S., Kunst F., Lenoble P., Meurice G., Sekowska A., Vallenet D., Wang T., Moszer I., Médigue C., others. 2009. From a consortium sequence to a unified sequence: the *Bacillus subtilis* 168 reference genome a decade later. *Microbiology* 155:1758–1775.

Beres SB., Sesso R., Pinto SWL., Hoe NP., Porcella SF., Deleo FR., Musser JM. 2008. Genome sequence of a Lancefield group C *Streptococcus zooepidemicus* strain causing epidemic nephritis: new information about an old disease. *PloS one* 3:e3026. DOI: 10.1371/journal.pone.0003026.

Beres SB., Sylva GL., Barbian KD., Lei B., Hoff JS., Mammarella ND., Liu M-Y., Smoot JC., Porcella SF., Parkins LD., Campbell DS., Smith TM., McCormick JK., Leung DYM., Schlievert PM., Musser JM. 2002. Genome sequence of a serotype M3 strain of group A *Streptococcus*: phage-encoded toxins, the high-virulence phenotype, and clone emergence. *Proceedings of the National Academy of Sciences of the United States of America* 99:10078–83. DOI: 10.1073/pnas.152298499.

Bessen DE., Kumar N., Hall GS., Riley DR., Luo F., Lizano S., Ford CN., McShan WM., Nguyen S V., Dunning Hotopp JC., Tettelin H. 2011. Whole-genome association study on tissue tropism phenotypes in group A *Streptococcus*. *Journal of bacteriology* 193:6651–63. DOI: 10.1128/JB.05263-11.

Biswas S., Biswas I. 2012. Complete genome sequence of *Streptococcus mutans* GS-5, a serotype c strain. *Journal of bacteriology* 194:4787–8. DOI: 10.1128/JB.01106-12.

Bolotin A., Quinquis B., Renault P., Sorokin A., Ehrlich SD., Kulakauskas S., Lapidus A., Goltsman E., Mazur M., Pusch GD., Fonstein M., Overbeek R., Kyprides N., Purnelle B., Prozzi D., Ngui K., Masuy D., Hancy F., Burteau S., Boutry M., Delcour J., Goffeau A., Hols P. 2004. Complete sequence and comparative genome analysis of the dairy bacterium *Streptococcus thermophilus*. *Nature biotechnology* 22:1554–8. DOI: 10.1038/nbt1034.

Boyle B., Vaillancourt K., Bonifait L., Charette SJ., Gottschalk M., Grenier D. 2012a. Genome sequence of the swine pathogen *Streptococcus suis* serotype 2 strain S735. *Journal of bacteriology* 194:6343–6344.

Boyle B., Vaillancourt K., Bonifait L., Charette SJ., Gottschalk M., Grenier D. 2012b. Genome sequence of the swine pathogen *Streptococcus suis* serotype 2 strain S735. *Journal of bacteriology* 194:6343–4. DOI: 10.1128/JB.01559-12.

Camilli R., Bonnal RJP., Del Grosso M., Iacono M., Corti G., Rizzi E., Marchetti M., Mulas L., Iannelli F., Superti F., others. 2011. Complete genome sequence of a serotype 11A, ST62 *Streptococcus pneumoniae* invasive isolate. *BMC microbiology* 11:25.

Chen C., Tang J., Dong W., Wang C., Feng Y., Wang J., Zheng F., Pan X., Liu D., Li M., Song Y., Zhu X., Sun H., Feng T., Guo Z., Ju A., Ge J., Dong Y., Sun W., Jiang Y., Wang J., Yan J., Yang H., Wang X., Gao GF., Yang R., Wang J., Yu J. 2007. A glimpse of streptococcal toxic shock syndrome from comparative genomics of *S. suis* 2 Chinese isolates. *PloS one* 2:e315. DOI: 10.1371/journal.pone.0000315.

Croucher NJ., Harris SR., Fraser C., Quail MA., Burton J., van der Linden M., McGee L., von Gottberg A., Song JH., Ko KS., others. 2011. Rapid pneumococcal evolution in response to clinical interventions. *science* 331:430–434.

Delorme C., Bartholini C., Luraschi M., Pons N., Loux V., Almeida M., Guédon E., Gibrat J-F., Renault P. 2011. Complete genome sequence of the pigmented *Streptococcus thermophilus* strain JIM8232. *Journal of bacteriology* 193:5581–2. DOI: 10.1128/JB.05404-11.

Denapaite D., Brückner R., Nuhn M., Reichmann P., Henrich B., Maurer P., Schähle Y., Selbmann P., Zimmermann W., Wambutt R., Hakenbeck R. 2010. The genome of *Streptococcus mitis* B6--what is a commensal? *PloS one* 5:e9426. DOI: 10.1371/journal.pone.0009426.

Ding F., Tang P., Hsu M-H., Cui P., Hu S., Yu J., Chiu C-H. 2009. Genome evolution driven by host adaptations results in a more virulent and antimicrobial-resistant *Streptococcus pneumoniae* serotype 14. *BMC genomics* 10:158. DOI: 10.1186/1471-2164-10-158.

Donati C., Hiller NL., Tettelin H., Muzzi A., Croucher NJ., Angiuoli S V., Oggioni M., Dunning Hotopp JC., Hu FZ., Riley DR., Covacci A., Mitchell TJ., Bentley SD., Kilian M., Ehrlich GD., Rappuoli R., Moxon ER., Masignani V. 2010. Structure and dynamics of the pan-genome of *Streptococcus pneumoniae* and closely related species. *Genome biology* 11:R107. DOI: 10.1186/gb-2010-11-10-r107.

Dopazo J., Mendoza A., Herrero J., Caldara F., Humbert Y., Friedli L., Guerrier M., Grand-Schenk E., Gandin C., de Francesco M., others. 2001. Annotated draft genomic sequence from a *Streptococcus pneumoniae* type 19F clinical isolate. *Microbial drug resistance* 7:99–125.

Ferretti JJ., McShan WM., Ajdic D., Savic DJ., Savic G., Lyon K., Primeaux C., Sezate S., Suvorov a N., Kenton S., Lai HS., Lin SP., Qian Y., Jia HG., Najar FZ., Ren Q., Zhu H., Song L., White J., Yuan X., Clifton SW., Roe B a., McLaughlin R. 2001. Complete genome sequence of an M1 strain of *Streptococcus pyogenes*. *Proceedings of the National Academy of Sciences of the*

United States of America 98:4658–63. DOI: 10.1073/pnas.071559398.

Fittipaldi N., Segura M., Grenier D., Gottschalk M. 2012. Virulence factors involved in the pathogenesis of the infection caused by the swine pathogen and zoonotic agent *Streptococcus suis*. *Future microbiology* 7:259–279.

Geng J., Chiu C-H., Tang P., Chen Y., Shieh H-R., Hu S., Chen Y-YM. 2012. Complete genome and transcriptomes of *Streptococcus parasanguinis* FW213: phylogenic relations and potential virulence mechanisms. *PLoS One* 7:e34769.

Geng J., Huang S-C., Li S., Hu S., Chen Y-YM. 2011. Complete genome sequence of the ureolytic *Streptococcus salivarius* strain 57.I. *Journal of bacteriology* 193:5596–7. DOI: 10.1128/JB.05670-11.

Glaser P., Rusniok C., Buchrieser C., Chevalier F., Frangeul L., Msadek T., Zouine M., Couvé E., Lalioui L., Poyart C., Trieu-Cuot P., Kunst F. 2002. Genome sequence of *Streptococcus agalactiae*, a pathogen causing invasive neonatal disease. *Molecular …* 45:1499–1513.

Green NM., Zhang S., Porcella SF., Nagiec MJ., Barbian KD., Beres SB., LeFebvre RB., Musser JM. 2005. Genome sequence of a serotype M28 strain of group A *Streptococcus*: potential new insights into puerperal sepsis and bacterial disease specificity. *The Journal of infectious diseases* 192:760–770.

Guédon E., Delorme C., Pons N., Cruaud C., Loux V., Couloux A., Gautier C., Sanchez N., Layec S., Galleron N., Almeida M., van de Guchte M., Kennedy SP., Ehrlich SD., Gibrat J-F., Wincker P., Renault P. 2011. Complete genome sequence of the commensal *Streptococcus salivarius* strain JIM8777. *Journal of bacteriology* 193:5024–5. DOI: 10.1128/JB.05390-11.

Holden MTG., Hauser H., Sanders M., Ngo TH., Cherevach I., Cronin A., Goodhead I., Mungall K., Quail M a., Price C., Rabbinowitsch E., Sharp S., Croucher NJ., Chieu TB., Mai NTH., Diep TS., Chinh NT., Kehoe M., Leigh J a., Ward PN., Dowson CG., Whatmore AM., Chanter N., Iversen P., Gottschalk M., Slater JD., Smith HE., Spratt BG., Xu J., Ye C., Bentley S., Barrell BG., Schultsz C., Maskell DJ., Parkhill J. 2009b. Rapid evolution of virulence and drug resistance in the emerging zoonotic pathogen *Streptococcus suis*. *PloS one* 4:e6072. DOI: 10.1371/journal.pone.0006072.

Holden MTG., Scott A., Cherevach I., Chillingworth T., Churcher C., Cronin A., Dowd L., Feltwell T., Hamlin N., Holroyd S., Jagels K., Moule S., Mungall K., Quail M a., Price C., Rabbinowitsch E., Sharp S., Skelton J., Whitehead S., Barrell BG., Kehoe M., Parkhill J. 2007. Complete genome of acute rheumatic fever-associated serotype M5 *Streptococcus pyogenes* strain manfredo. *Journal of bacteriology* 189:1473–7. DOI: 10.1128/JB.01227-06.

Hoskins J., Alborn W., Arnold J., Blaszack L., Burgett S., DeHoff B., Estrem S., Fritz L., Al. E. 2001. Genome of the Bacterium *Streptococcus pneumoniae* Strain R6. *Journal of bacteriology* 183:5709–5717. DOI: 10.1128/JB.183.19.5709.

Hu P., Yang M., Zhang A., Wu J., Chen B., Hua Y., Yu J., Chen H., Xiao J., Jin M. 2011. Complete genome sequence of *Streptococcus suis* serotype 3 strain ST3. *Journal of bacteriology*

193:3428–9. DOI: 10.1128/JB.00083-11.

Jans C., Gerber A., Bugnard J., Njage PMK., Lacroix C., Meile L. 2012. Novel Streptococcus infantarius subsp. infantarius variants harboring lactose metabolism genes homologous to *Streptococcus thermophilus*. *Food microbiology* 31:33–42.

Jin D., Chen C., Li L., Lu S., Li Z., Zhou Z., Jing H., Xu Y., Du P., Wang H., others. 2013. Dynamics of fecal microbial communities in children with diarrhea of unknown etiology and genomic analysis of associated *Streptococcus lutetiensis*. *BMC microbiology* 13:141.

Kang X., Ling N., Sun G., Zhou Q., Zhang L., Sheng Q. 2012. Complete genome sequence of *Streptococcus thermophilus* strain MN-ZLW-002. *Journal of bacteriology* 194:4428–4429.

Kunst F., Ogasawara N., Moszer I., Albertini AM., Alloni GO., Azevedo V., Bertero MG., Bessieres P., Bolotin A., Borchert S., others. 1997. The complete genome sequence of the gram-positive bacterium *Bacillus subtilis*. *Nature* 390:249.

Lanie J a., Ng W-L., Kazmierczak KM., Andrzejewski TM., Davidsen TM., Wayne KJ., Tettelin H., Glass JI., Winkler ME. 2007. Genome sequence of Avery's virulent serotype 2 strain D39 of *Streptococcus pneumoniae* and comparison with that of unencapsulated laboratory strain R6. *Journal of bacteriology* 189:38–51. DOI: 10.1128/JB.01148-06.

Li G., Hu FZ., Yang X., Cui Y., Yang J., Qu F., Gao GF., Zhang J-R. 2012. Complete genome sequence of *Streptococcus pneumoniae* strain ST556, a multidrug-resistant isolate from an otitis media patient. *Journal of bacteriology* 194:3294–3295.

Lin I-H., Liu T-T., Teng Y-T., Wu H-L., Liu Y-M., Wu K-M., Chang C-H., Hsu M-T. 2011. Sequencing and comparative genome analysis of two pathogenic *Streptococcus gallolyticus* subspecies: genome plasticity, adaptation and virulence. *PloS one* 6:e20519. DOI: 10.1371/journal.pone.0020519.

Liu G., Zhang W., Lu C. 2012. Complete genome sequence of *Streptococcus agalactiae* GD201008-001, isolated in China from tilapia with meningoencephalitis. *Journal of bacteriology* 194:6653.

Ma Z., Geng J., Zhang H., Yu H., Yi L., Lei M., Lu C., Fan H., Hu S. 2011. Complete genome sequence of *Streptococcus equi* subsp. zooepidemicus strain ATCC 35246. *Journal of bacteriology* 193:5583–5584.

Makarova K., Slesarev a., Wolf Y., Sorokin a., Mirkin B., Koonin E., Pavlov a., Pavlova N., Karamychev V., Polouchine N., Shakhova V., Grigoriev I., Lou Y., Rohksar D., Lucas S., Huang K., Goodstein DM., Hawkins T., Plengvidhya V., Welker D., Hughes J., Goh Y., Benson a., Baldwin K., Lee J-H., Díaz-Muñiz I., Dosti B., Smeianov V., Wechter W., Barabote R., Lorca G., Altermann E., Barrangou R., Ganesan B., Xie Y., Rawsthorne H., Tamir D., Parker C., Breidt F., Broadbent J., Hutkins R., O'Sullivan D., Steele J., Unlu G., Saier M., Klaenhammer T., Richardson P., Kozyavkin S., Weimer B., Mills D. 2006. Comparative genomics of the lactic acid bacteria. *Proceedings of the National Academy of Sciences of the United States of America* 103:15611–6. DOI: 10.1073/pnas.0607117103.

Maruyama F., Kobata M., Kurokawa K., Nishida K., Sakurai A., Nakano K., Nomura R., Kawabata S., Ooshima T., Nakai K., Hattori M., Hamada S., Nakagawa I. 2009. Comparative genomic analyses of *Streptococcus mutans* provide insights into chromosomal shuffling and species-specific content. *BMC genomics* 10:358. DOI: 10.1186/1471-2164-10-358.

McShan WM., Ferretti JJ., Karasawa T., Suvorov AN., Lin S., Qin B., Jia H., Kenton S., Najar F., Wu H., Scott J., Roe B a., Savic DJ. 2008. Genome sequence of a nephritogenic and highly transformable M49 strain of *Streptococcus pyogenes*. *Journal of bacteriology* 190:7773–85. DOI: 10.1128/JB.00672-08.

Miyoshi-Akiyama T., Watanabe S., Kirikae T. 2012. Complete genome sequence of *Streptococcus pyogenes* M1 476, isolated from a patient with streptococcal toxic shock syndrome. *Journal of bacteriology* 194:5466.

Nakagawa I., Kurokawa K., Yamashita A., Nakata M., Tomiyasu Y., Okahashi N., Kawabata S., Yamazaki K., Shiba T., Yasunaga T., Hayashi H., Hattori M., Hamada S. 2003. Genome Sequence of an M3 Strain of *Streptococcus pyogenes* Reveals a Large-Scale Genomic Rearrangement in Invasive Strains and New Insights into Phage Evolution. *Genome Research* 13:1042–1055. DOI: 10.1101/gr.1096703.1.

Nho SW., Hikima J., Cha IS., Park S Bin., Jang H Bin., del Castillo CS., Kondo H., Hirono I., Aoki T., Jung TS. 2011. Complete genome sequence and immunoproteomic analyses of the bacterial fish pathogen *Streptococcus parauberis*. *Journal of bacteriology* 193:3356–66. DOI: 10.1128/JB.00182-11.

Okumura K., Shimomura Y., Murayama SY., Yagi J., Ubukata K., Kirikae T., Miyoshi-Akiyama T. 2012. Evolutionary paths of streptococcal and staphylococcal superantigens. *BMC genomics* 13:404.

Papadimitriou K., Ferreira S., Papandreou NC., Mavrogonatou E., Supply P., Pot B., Tsakalidou E. 2012. Complete genome sequence of the dairy isolate *Streptococcus macedonicus* ACA-DC 198. *Journal of bacteriology* 194:1838–9. DOI: 10.1128/JB.06804-11.

Pereira UDP., Rodrigues Dos Santos A., Hassan SS., Aburjaile FF., Soares SDC., Ramos RTJ., Carneiro AR., Guimarães LC., Silva de Almeida S., Diniz CAA., Barbosa MS., Gomes de Sá P., Ali A., Bakhtiar SM., Dorella FA., Zerlotini A., Araújo FMG., Leite LR., Oliveira G., Miyoshi A., Silva A., Azevedo V., Figueiredo HCP. 2013. Complete genome sequence of *Streptococcus agalactiae* strain SA20-06, a fish pathogen associated to meningoencephalitis outbreaks. *Standards in genomic sciences* 8:188–97. DOI: 10.4056/sigs.3687314.

Port GC., Paluscio E., Caparon MG. 2013. Complete genome sequence of emm type 14 Streptococcus pyogenes strain HSC5. *Genome announcements* 1:e00612--13.

Rachinger M., Volland S., Meinhardt F., Daniel R., Liesegang H. 2013. First insights into the completely annotated genome sequence of *Bacillus licheniformis* strain 9945A. *Genome announcements* 1:e00525--13.

Reichmann P., Nuhn M., Denapaite D., Brückner R., Henrich B., Maurer P., Rieger M., Klages S., Reinhard R., Hakenbeck R. 2011. Genome of *Streptococcus oralis* strain Uo5. *Journal of*

bacteriology 193:2888–9. DOI: 10.1128/JB.00321-11.

Rosinski-Chupin, I., Sauvage, E., Mairey, B., Mangenot, S., Ma, L., Da Cunha, V., ... & Glaser, P. (2013). Reductive evolution in *Streptococcus agalactiae* and the emergence of a host adapted l ineage. *BMC genomics*. *14*(1), 252.

Rusniok C., Couvé E., Da Cunha V., El Gana R., Zidane N., Bouchier C., Poyart C., Leclercq R., Trieu-Cuot P., Glaser P. 2010. Genome sequence of *Streptococcus gallolyticus*: insights into its adaptation to the bovine rumen and its ability to cause endocarditis. *Journal of bacteriology* 192:2266–76. DOI: 10.1128/JB.01659-09.

Shahinas D., Tamber GS., Arya G., Wong A., Lau R., Jamieson F., Ma JH., Alexander DC., Low DE., Pillai DR. 2011. Whole-genome sequence of *Streptococcus pseudopneumoniae* isolate IS7493. *Journal of bacteriology* 193:6102–3. DOI: 10.1128/JB.06075-11.

Shimomura Y., Okumura K., Murayama SY., Yagi J., Ubukata K., Kirikae T., Miyoshi-Akiyama T. 2011. Complete genome sequencing and analysis of a Lancefield group G *Streptococcus dysgalactiae* subsp. equisimilis strain causing streptococcal toxic shock syndrome (STSS). *BMC genomics* 12:17. DOI: 10.1186/1471-2164-12-17.

Sumby P., Porcella S., Madrigal A., Barbian K., Virtaneva K., Ricklefs S., Sturdevant D., Graham M. 2005. Evolutionary Origin and Emergence of a Highly Successful Clone of Serotype M1 Group A. *The Journal of Infectious Disease* 192:771–782.

Sun Z., Chen X., Wang J., Zhao W., Shao Y., Wu L., Zhou Z., Sun T., Wang L., Meng H., others. 2011. Complete genome sequence of *Streptococcus thermophilus* strain ND03. *Journal of bacteriology* 193:793–794.

Suzuki H., Lefébure T., Hubisz MJ., Pavinski Bitar P., Lang P., Siepel A., Stanhope MJ. 2011. Comparative genomic analysis of the *Streptococcus dysgalactiae* species group: gene content, molecular adaptation, and promoter evolution. *Genome biology and evolution* 3:168–85. DOI: 10.1093/gbe/evr006.

Tomoyasu T., Tabata A., Imaki H., Tsuruno K., Miyazaki A., Sonomoto K., Whiley RA., Nagamune H. 2012. Role of *Streptococcus intermedius* DnaK chaperone system in stress tolerance and pathogenicity. *Cell stress and chaperones* 17:41–55.

Vickerman MM., Iobst S., Jesionowski a M., Gill SR. 2007. Genome-wide transcriptional changes in *Streptococcus gordonii* in response to competence signaling peptide. *Journal of bacteriology* 189:7799–807. DOI: 10.1128/JB.01023-07.

Wang H., Yuan R., Chai Y., Cao Y., Gan X., Chen Y., Wang Y. 2013. An ultrasensitive peroxydisulfate electrochemiluminescence immunosensor for *Streptococcus suis* serotype 2 based on l-cysteine combined with mimicking bi-enzyme synergetic catalysis to in situ generate coreactant. *Biosensors and Bioelectronics* 43:63–68.

Ward PN., Holden MTG., Leigh J a., Lennard N., Bignell A., Barron A., Clark L., Quail M a., Woodward J., Barrell BG., Egan S a., Field TR., Maskell D., Kehoe M., Dowson CG., Chanter N., Whatmore

AM., Bentley SD., Parkhill J. 2009. Evidence for niche adaptation in the genome of the bovine pathogen *Streptococcus uberis*. *BMC genomics* 10:54. DOI: 10.1186/1471-2164-10-54.

Watanabe S., Kirikae T., Miyoshi-Akiyama T. 2013. Complete genome sequence of Streptococcus dysgalactiae subsp. equisimilis 167 carrying Lancefield group C antigen and comparative genomics of *S. dysgalactiae* subsp. equisimilis strains. *Genome biology and evolution* 5:1644–51. DOI: 10.1093/gbe/evt117.

Wu Z., Wang W., Tang M., Shao J., Dai C., Zhang W., Fan H., Yao H., Zong J., Chen D., others. 2014. Comparative genomic analysis shows that *Streptococcus suis* meningitis isolate SC070731 contains a unique 105K genomic island. *Gene* 535:156–164.

Xu P., Alves JM., Kitten T., Brown A., Chen Z., Ozaki LS., Manque P., Ge X., Serrano MG., Puiu D., Hendricks S., Wang Y., Chaplin MD., Akan D., Paik S., Peterson DL., Macrina FL., Buck G a. 2007. Genome of the opportunistic pathogen *Streptococcus sanguinis*. *Journal of bacteriology* 189:3166–75. DOI: 10.1128/JB.01808-06.

Ye C., Zheng H., Zhang J., Jing H., Wang L., Xiong Y., Wang W., Zhou Z., Sun Q., Luo X., others. 2009. Clinical, experimental, and genomic differences between intermediately pathogenic, highly pathogenic, and epidemic S*treptococcus suis*. *The Journal of infectious diseases* 199:97–107.

Zhang A., Yang M., Hu P., Wu J., Chen B., Hua Y., Yu J., Chen H., Xiao J., Jin M. 2011. Comparative genomic analysis of *Streptococcus suis* reveals significant genomic diversity among different serotypes. *BMC genomics* 12:523.

Zhang B., Zhang J., Sun L. 2014. *Streptococcus iniae* SF1: complete genome sequence, proteomic profile, and immunoprotective antigens. *PloS one* 9:e91324.

Zheng P-X., Chung K-T., Chiang-Ni C., Wang S-Y., Tsai P-J., Chuang W-J., Lin Y-S., Liu C-C., Wu J-J. 2013. Complete genome sequence of emm1 *Streptococcus pyogenes* A20, a strain with an intact two-component system, CovRS, isolated from a patient with necrotizing fasciitis. *Genome announcements* 1:e00149--12.

Zubair S., De Villiers EP., Fuxelius HH., Andersson G., Johansson K-E., Bishop RP., Bongcam-Rudloff E. 2013. Genome sequence of *Streptococcus agalactiae* strain 09mas018883, isolated from a Swedish cow. *Genome announcements* 1:e00456--13.
