# Supplementary material for: Global genomic similarity and core genome sequence diversity of the Streptococcus genus as a toolkit to identify closely related bacterial species in complex environments
Source: PeerJ. 2019 Jan 14;6:e6233. doi: 10.7717/peerj.6233 (PMC6336011; doi:10.7717/peerj.6233)
Supplement: Supplemental Information 2 [file peerj-07-6233-s002.pdf]

Position	Sequence ID	Genus Average identity	SD	Accession (S. pyogenes str. Manfredo)	Annotation
1	59	99.56	0.77	YP_001127646.1	30S ribosomal protein S19
2	72	98.58	1.32	YP_001127667.1	30S ribosomal protein S11
3	304	98.08	1.37	YP_001128728.1	50S ribosomal protein L20
4	54	97.93	1.81	YP_001127641.1	30S ribosomal protein S10
5	62	97.89	1.83	YP_001127649.1	50S ribosomal protein L16
6	103	97.49	2.18	YP_001129139.1	50S ribosomal protein L13
7	63	97.37	2.47	YP_001127652.1	50S ribosomal protein L14
8	61	97.11	2.41	YP_001127648.1	30S ribosomal protein S3
9	396	97.1	1.80	YP_001129319.1	30S ribosomal protein S4
10	298	96.6	2.57	YP_001128714.1	50S ribosomal protein L27
11	65	96.43	2.64	YP_001127654.1	50S ribosomal protein L5
12	60	96.23	4.74	YP_001127647.1	50S ribosomal protein L22
13	368	96.13	2.31	YP_001127805.1	elongation factor G
14	58	96.11	3.71	YP_001127645.1	50S ribosomal protein L2
15	55	96.01	2.75	YP_001127642.1	50S ribosomal protein L3
16	147	95.77	2.11	YP_001128896.1	elongation factor Tu
17	76	95.77	2.14	YP_001127937.1	molecular chaperone DnaK
18	68	95.73	2.57	YP_001127659.1	30S ribosomal protein S5
19	366	95.45	2.79	YP_001127843.1	phosphoglycerate kinase
20	106	95.45	2.89	YP_001128168.1	BipA family GTPase
21	2	95.24	1.96	YP_001127607.1	GTP-dependent nucleic acid-binding protein EngD
22	370	95.19	3.29	YP_001127803.1	30S ribosomal protein S12
23	369	95.19	3.65	YP_001127804.1	30S ribosomal protein S7
24	57	95.11	4.89	YP_001127644.1	50S ribosomal protein L23
25	167	94.98	2.97	YP_001128766.1	F0F1 ATP synthase subunit beta
26	126	94.95	3.93	YP_001128787.1	phosphopyruvate hydratase
27	73	94.89	4.30	YP_001127668.1	DNA-directed RNA polymerase subunit alpha
28	97	94.82	3.29	YP_001127676.1	DNA-directed RNA polymerase subunit beta
29	66	94.31	3.60	YP_001127656.1	30S ribosomal protein S8
30	56	94.21	4.97	YP_001127643.1	50S ribosomal protein L4
31	358	94.07	4.02	YP_001127886.1	30S ribosomal protein S6
32	372	93.96	3.13	YP_001127727.1	adenylosuccinate synthetase
33	166	93.88	4.30	YP_001128768.1	F0F1 ATP synthase subunit alpha
34	98	93.88	3.84	YP_001127677.1	DNA-directed RNA polymerase subunit beta'
35	404	93.78	3.61	YP_001129342.1	inosine 5'-monophosphate dehydrogenase
36	43	93.72	3.59	YP_001127754.1	queuine tRNA-ribosyltransferase
37	211	93.66	3.68	YP_001129345.1	ABC transporter ATP-binding protein
38	253	93.58	3.44	YP_001128484.1	peptide chain release factor 1
39	319	93.44	4.72	YP_001129003.1	GTP-binding protein Era
40	64	93.37	3.73	YP_001127653.1	50S ribosomal protein L24
41	325	93.11	4.58	YP_001129053.1	ATP-dependent Clp protease proteolytic subunit
42	305	92.89	6.45	YP_001128730.1	translation initiation factor IF-3
43	90	92.49	4.69	YP_001127818.1	hypothetical protein SpyM50224
44	399	92.14	3.32	YP_001129325.1	tRNA-specific 2-thiouridylase MnmA
45	69	91.98	4.40	YP_001127661.1	50S ribosomal protein L15
46	383	91.94	3.87	YP_001129237.1	chaperonin GroEL
47	336	91.92	4.02	YP_001129091.1	GTP-binding protein EngA
48	262	91.58	5.19	YP_001128642.1	30S ribosomal protein S1
49	237	91.17	5.33	YP_001128438.1	GMP synthase
50	149	91.17	3.94	YP_001128270.1	recombination protein RecR
51	28	91.01	3.84	YP_001127948.1	(3R)-hydroxymyristoyl-ACP dehydratase
52	367	90.81	5.90	YP_001127806.1	glyceraldehyde-3-phosphate dehydrogenase
53	388	90.8	5.38	YP_001129272.1	recombinase A
54	387	90.61	6.50	YP_001129271.1	Spx family transcriptional regulator
55	373	90.48	5.83	YP_001129255.1	elongation factor Ts
56	265	90.01	5.04	YP_001128647.1	DNA topoisomerase IV subunit B
57	174	89.91	5.53	YP_001128551.1	phosphoglucosamine mutase
58	230	89.86	5.47	YP_001128909.1	lysyl-tRNA synthetase
59	14	89.83	6.38	YP_001128037.1	DNA-directed RNA polymerase subunit omega
60	242	89.77	6.21	YP_001128404.1	phosphate ABC transporter permease
61	133	89.58	6.78	YP_001128956.1	response regulator protein
62	320	89.4	6.33	YP_001129013.1	ribosome recycling factor
63	13	89.36	5.56	YP_001128036.1	guanylate kinase
64	67	89.33	5.52	YP_001127657.1	50S ribosomal protein L6
65	159	89.29	5.70	YP_001128320.1	methionine aminopeptidase
66	86	89.09	5.60	YP_001127814.1	ABC transporter ATP-binding protein
67	238	89.08	5.95	YP_001128442.1	signal recognition particle protein
68	84	88.96	6.15	YP_001127837.1	fructose-bisphosphate aldolase
69	179	88.88	7.07	YP_001128372.1	pyruvate kinase
70	364	88.82	5.25	YP_001127846.1	glutamine synthetase
71	234	88.82	5.67	YP_001128532.1	tRNA modification GTPase TrmE
72	130	88.69	8.96	YP_001128973.1	catabolite control protein A
73	346	88.65	6.60	YP_001127928.1	aspartyl/glutamyl-tRNA amidotransferase subunit A
74	169	88.64	7.74	YP_001128761.2	phenylalanyl-tRNA synthetase subunit alpha
75	144	88.6	7.06	YP_001128912.1	peptidase
76	220	88.53	5.92	YP_001127956.1	mannose-specific phosphotransferase system (PTS), IIAB component
77	333	88.42	6.75	YP_001129085.1	transcription elongation factor GreA
78	236	88.18	7.96	YP_001128474.1	L-lactate dehydrogenase
79	226	87.74	7.10	YP_001127674.1	tyrosyl-tRNA synthetase

80	280	87.55	6.58	YP_001128530.1	50S ribosomal protein L10
81	386	87.48	6.44	YP_001129266.1	anaerobic ribonucleoside triphosphate reductase
82	239	87.36	5.18	YP_001128441.1	DNA-binding protein
83	36	87.33	6.66	YP_001127969.1	transcription elongation factor NusA
84	394	87.09	6.98	YP_001129303.1	aspartyl-tRNA synthetase
85	206	87.04	8.60	YP_001127697.1	tRNA-dihydrouridine synthase
86	326	86.96	6.55	YP_001129071.1	ribosomal large subunit pseudouridine synthase B
87	223	86.96	6.31	YP_001127769.1	NAD(P)H-dependent glycerol-3-phosphate dehydrogenase
88	148	86.89	9.57	YP_001128895.1	triosephosphate isomerase
89	345	86.61	7.38	YP_001129116.1	GTP-binding protein YqeH
90	263	86.41	7.35	YP_001128644.1	branched-chain amino acid aminotransferase
91	274	86.31	6.12	YP_001128629.1	adenine phosphoribosyltransferase
92	77	86.31	7.01	YP_001127938.1	chaperone protein DnaJ
93	156	86.18	7.91	YP_001128308.1	uridine kinase
94	375	86.14	6.81	YP_001129164.1	prolyl-tRNA synthetase
95	241	86.02	7.76	YP_001128406.1	phosphate transporter ATP-binding protein
96	222	85.91	5.75	YP_001127768.1	UTP--glucose-1-phosphate uridylyltransferase 2
97	50	85.87	5.59	YP_001127634.1	Holliday junction DNA helicase RuvB
98	381	85.81	7.71	YP_001129175.1	GTP pyrophosphokinase
99	40	85.79	8.24	YP_001127973.1	ribosome-binding factor A
100	35	85.78	6.75	YP_001127967.1	tRNA (guanine-N(7)-)-methyltransferase
101	183	85.64	6.49	YP_001128473.1	DNA gyrase subunit A
102	356	85.54	11.11	YP_001127890.1	CorA-like Mg2+ transporter protein
103	196	85.47	7.13	YP_001129044.1	exodeoxyribonuclease
104	371	85.45	8.27	YP_001127801.1	pur operon repressor
105	158	85.34	6.55	YP_001128316.1	UDP-N-acetylglucosamine 1-carboxyvinyltransferase
106	349	85.33	7.43	YP_001127903.1	preprotein translocase subunit SecA
107	281	85.32	6.41	YP_001128665.1	ribosome biogenesis GTP-binding protein YsxC
108	154	85.27	8.49	YP_001128283.1	superoxide dismutase
109	397	85.16	9.08	YP_001129321.1	replicative DNA helicase
110	113	85.13	8.05	YP_001128180.1	isoleucyl-tRNA synthetase
111	180	85.09	7.66	YP_001128374.1	glucosamine--fructose-6-phosphate aminotransferase
112	37	85.03	8.04	YP_001127970.1	hypothetical protein SpyM50380
113	120	84.99	7.53	YP_001128873.1	cell division ATP-binding protein
114	18	84.85	9.33	YP_001128045.1	response regulator protein
115	357	84.81	9.25	YP_001127887.1	single-stranded DNA-binding protein
116	23	84.79	6.94	YP_001127943.1	enoyl-ACP reductase
117	70	84.71	10.34	YP_001127662.1	preprotein translocase subunit SecY
118	250	84.69	6.78	YP_001128430.1	formate--tetrahydrofolate ligase
119	315	84.64	7.25	YP_001128982.1	SsrA-binding protein
120	182	84.63	9.81	YP_001128475.1	NADH oxidase
121	292	84.61	7.25	YP_001128694.1	tRNA (guanine-N(1)-)-methyltransferase
122	258	84.59	8.50	YP_001128518.1	spermidine/putrescine ABC transporter permease
123	395	84.44	7.91	YP_001129304.1	histidyl-tRNA synthetase
124	219	84.43	10.16	YP_001127954.1	PTS system mannose-specific transporter subunit IID
125	71	84.4	7.98	YP_001127663.1	adenylate kinase
126	151	84.38	8.72	YP_001128275.1	DEAD/DEAH box helicase
127	334	84.29	6.74	YP_001129088.1	UDP-N-acetylmuramate--L-alanine ligase
128	30	84.27	9.13	YP_001127951.1	acetyl-CoA carboxylase subunit alpha
129	12	84.25	7.67	YP_001128027.1	RNA methylase family protein
130	363	84.22	6.77	YP_001127851.1	DNA-binding/iron metalloprotein/AP endonuclease
131	139	84.22	9.37	YP_001128918.1	HPr kinase/phosphorylase
132	254	84.19	9.69	YP_001128485.1	thymidine kinase
133	29	84.11	8.91	YP_001127950.1	acetyl-CoA carboxylase subunit beta
134	374	84.1	9.20	YP_001129163.1	DNA polymerase III PolC
135	276	84.09	8.53	YP_001128632.1	ribonuclease Z
136	283	84.08	6.55	YP_001128669.1	thymidylate synthase
137	344	84.01	8.42	YP_001129114.1	nicotinic acid mononucleotide adenylyltransferase
138	191	84.01	7.72	YP_001128657.1	LysR family transcriptional regulator
139	309	83.94	8.28	YP_001129062.1	manganese-dependent inorganic pyrophosphatase
140	359	83.74	8.43	YP_001127882.1	thioredoxin
141	205	83.73	9.25	YP_001127698.1	Hsp33-like chaperonin
142	248	83.62	6.67	YP_001128654.1	orotate phosphoribosyltransferase
143	270	83.6	9.63	YP_001128572.1	7,8-dihydro-8-oxoguanine-triphosphatase
144	102	83.5	6.85	YP_001129144.1	SpoU rRNA methylase family protein
145	260	83.4	8.46	YP_001128382.1	DNA helicase II
146	95	83.31	8.26	YP_001127672.1	ABC transporter ATP-binding protein
147	257	83.28	9.00	YP_001128517.1	spermidine/putrescine ABC transporter permease
148	82	83.27	7.60	YP_001127833.1	trigger factor
149	294	83.26	10.18	YP_001128710.1	bifunctional pyrimidine regulatory protein PyrR/uracil phosphoribosyltransferase
150	244	83.08	11.60	YP_001128398.1	Spx family transcriptional regulator
151	264	82.84	8.90	YP_001128645.1	DNA topoisomerase IV subunit A
152	83	82.84	8.10	YP_001127774.1	deoxyuridine 5'-triphosphate nucleotidohydrolase
153	123	82.79	8.96	YP_001128865.1	hypothetical protein SpyM51324
154	266	82.74	9.40	YP_001128564.1	Zn-dependent hydrolase
155	398	82.59	9.32	YP_001129322.1	50S ribosomal protein L9
156	25	82.46	8.84	YP_001127945.1	3-ketoacyl-ACP reductase
157	311	82.33	9.47	YP_001129064.1	transporter
158	347	82.3	10.64	YP_001127927.1	aspartyl/glutamyl-tRNA amidotransferase subunit C
159	122	82.26	7.82	YP_001128866.1	hypothetical protein SpyM51325
160	240	82.24	11.62	YP_001128407.1	phosphate transport system protein
161	175	82.01	9.33	YP_001128549.1	coproporphyrinogen III oxidase

162	299	81.94	7.73	YP_001128718.1	thiamine biosynthesis protein ThiI
163	177	81.69	10.26	YP_001128547.1	haloacid dehalogenase-like hydrolase
164	26	81.63	10.15	YP_001127946.1	3-oxoacyl-(acyl carrier protein) synthase II
165	293	81.47	9.41	YP_001128695.1	16S rRNA-processing protein RimM
166	295	81.29	8.36	YP_001128711.1	RNA pseudouridylate synthase
167	390	81.15	10.37	YP_001129275.1	Holliday junction DNA helicase RuvA
168	354	81.13	18.45	YP_001127893.1	cytidine and deoxycytidylate deaminase
169	227	80.87	9.67	YP_001128264.1	dihydroorotate dehydrogenase 1A
170	42	80.84	8.48	YP_001127749.1	DNA polymerase I
171	171	80.79	8.66	YP_001128561.1	ABC transporter ATP-binding protein
172	109	80.67	11.18	YP_001128173.1	cell division protein
173	310	80.57	10.34	YP_001129063.1	pyruvate formate-lyase activating enzyme
174	255	80.51	11.97	YP_001128497.1	inorganic polyphosphate/ATP-NAD kinase
175	7	80.45	9.29	YP_001127995.1	glycyl-tRNA synthetase subunit beta
176	259	80.4	10.28	YP_001128520.1	UDP-N-acetylenolpyruvoylglucosamine reductase
177	313	80.38	8.44	YP_001128747.1	hypothetical protein SpyM51206
178	279	80.34	10.98	YP_001128533.1	dipeptidase PepV
179	378	80.25	10.06	YP_001129167.1	undecaprenyl pyrophosphate synthase
180	87	80.18	9.17	YP_001127815.1	hypothetical protein SpyM50221
181	1	80.1	13.94	YP_001127604.1	chromosomal replication initiation protein
182	342	80.07	11.75	YP_001129111.1	hypothetical protein SpyM51588
183	306	80.02	11.43	YP_001128731.1	cytidylate kinase
184	380	79.95	10.26	YP_001129174.1	D-tyrosyl-tRNA(Tyr) deacylase
185	207	79.88	9.33	YP_001129160.1	peptide deformylase
186	96	79.79	10.32	YP_001127673.1	ABC transporter permease
187	391	79.74	10.11	YP_001129277.1	DNA mismatch repair protein
188	150	79.59	8.58	YP_001128271.1	D-alanyl-alanine synthetase A
189	190	79.57	11.63	YP_001128503.1	redox-sensing transcriptional repressor Rex
190	33	79.55	9.49	YP_001127964.1	ABC transporter ATP-binding protein
191	247	79.46	10.79	YP_001128649.1	dihydroorotase
192	290	79.4	8.24	YP_001128689.1	1-phosphofructokinase
193	307	79.31	10.88	YP_001128735.1	peptidase T
194	198	79.2	8.71	YP_001129100.1	16S rRNA methyltransferase GidB
195	215	79.18	9.68	YP_001128024.1	Holliday junction-specific endonuclease
196	101	79.1	9.52	YP_001129146.1	hypothetical protein SpyM51627
197	193	79.09	10.84	YP_001128469.1	hypothetical protein SpyM50910
198	143	79.03	12.44	YP_001128913.1	peptidase
199	353	79.01	13.70	YP_001127895.1	hypothetical protein SpyM50304
200	400	78.91	11.59	YP_001129329.1	cobalt transport protein
201	243	78.73	11.61	YP_001128402.1	phosphate ABC transporter,extracellular phosphate-binding lipoprotein
202	79	78.61	8.27	YP_001127828.1	tRNA pseudouridine synthase A
203	125	78.59	12.79	YP_001128795.1	flavodoxin
204	267	78.48	9.61	YP_001128566.1	ABC transporter permease
205	331	78.38	8.98	YP_001129078.1	glutamate racemase
206	200	78.2	9.18	YP_001127921.1	L-asparaginase
207	155	78.2	9.54	YP_001128291.1	O-methyltransferase
208	117	78.14	11.59	YP_001128196.1	DNA repair protein
209	88	78.13	8.31	YP_001127816.1	cysteine desulfurase
210	38	78.08	12.10	YP_001127971.1	hypothetical protein SpyM50381
211	256	78.05	13.32	YP_001128516.1	spermidine/putrescine extracellular binding protein
212	360	77.98	11.68	YP_001127880.1	DNA mismatch repair protein
213	75	77.9	11.78	YP_001127936.1	heat shock protein GrpE
214	362	77.88	12.19	YP_001127858.1	DNA-binding protein
215	114	77.87	11.70	YP_001128191.1	exodeoxyribonuclease VII large subunit
216	134	77.83	13.26	YP_001128955.1	sensor histidine kinase
217	351	77.78	9.51	YP_001127901.1	fructokinase
218	194	77.73	9.85	YP_001128349.1	glutamine ABC transporter,glutamine-binding protein/permease protein
219	393	77.72	12.78	YP_001129302.1	hypothetical protein SpyM51787
220	168	77.65	14.79	YP_001128765.1	F0F1 ATP synthase subunit epsilon
221	39	77.63	9.26	YP_001127972.1	translation initiation factor IF-2
222	74	77.49	13.08	YP_001127935.1	heat-inducible transcription repressor
223	189	77.4	10.21	YP_001128502.1	hypothetical protein SpyM50945
224	9	77.33	10.96	YP_001128013.1	phospho-N-acetylmuramoyl-pentapeptide-transferase
225	152	77.29	12.41	YP_001128279.1	1-acyl-sn-glycerol-3-phosphate acyltransferase
226	316	77.22	11.95	YP_001128983.1	exoribonuclease R
227	340	77.19	12.78	YP_001129105.1	ABC transporter permease
228	135	76.99	11.03	YP_001128953.1	ribonuclease III
229	202	76.94	14.08	YP_001127897.1	sucrose operon repressor
230	350	76.82	11.08	YP_001127902.1	mannose-6-phosphate isomerase
231	121	76.67	9.55	YP_001128868.1	aspartate aminotransferase
232	10	76.67	13.06	YP_001128015.1	ABC transporter permease
233	21	76.53	14.43	YP_001127940.1	MarR family transcriptional regulator
234	229	76.52	13.74	YP_001128923.1	hypothetical protein SpyM51383
235	161	76.49	9.14	YP_001128774.1	NAD-dependent DNA ligase LigA
236	162	76.24	20.85	YP_001128773.1	lipid kinase
237	301	76.1	9.31	YP_001128724.1	chorismate synthase
238	107	75.98	9.61	YP_001128170.1	UDP-N-acetylmuramoyl-L-alanyl-D-glutamate synthetase
239	321	75.97	10.18	YP_001129017.1	DNA translocase FtsK
240	249	75.93	10.13	YP_001128655.1	orotidine 5'-phosphate decarboxylase
241	377	75.62	13.17	YP_001129166.1	phosphatidate cytidylyltransferase
242	297	75.31	12.69	YP_001128713.1	LysR family transcriptional regulator
243	146	75.01	13.22	YP_001128898.1	cell division protein

244	157	74.88	12.56	YP_001128311.1	hypothetical protein SpyM50747
245	291	74.68	13.86	YP_001128693.1	pyridine nucleotide-disulfide oxidoreductase family protein
246	15	74.62	12.67	YP_001128040.1	16S rRNA m(5)C 967 methyltransferase
247	382	74.61	12.42	YP_001129180.1	16S ribosomal RNA methyltransferase RsmE
248	16	74.56	14.93	YP_001128041.1	protein phosphatase
249	401	74.41	11.01	YP_001129331.1	cobalt transporter ATP-binding subunit
250	376	74.38	11.70	YP_001129165.1	pheromone-processing membrane metalloprotease
251	145	74.36	10.86	YP_001128899.1	phosphoenolpyruvate carboxylase
252	118	74.28	11.77	YP_001128198.1	hypothetical protein SpyM50627
253	235	74.18	11.92	YP_001128412.1	pantothenate kinase
254	322	74.12	11.89	YP_001129026.1	ADP-ribose pyrophosphatase
255	3	74.08	11.86	YP_001127608.1	peptidyl-tRNA hydrolase
256	216	74.06	9.95	YP_001128029.2	S-ribosylhomocysteinase
257	343	73.79	13.71	YP_001129113.1	hypothetical protein SpyM51590
258	100	73.64	13.95	YP_001127686.1	hypothetical protein SpyM50091
259	6	73.57	14.68	YP_001127991.1	N-acetylglucosamine-6-phosphate deacetylase
260	221	73.45	13.04	YP_001127963.1	HIT-family protein
261	108	73.45	10.62	YP_001128171.1	undecaprenyldiphospho-muramoylpentapeptide beta-N- acetylglucosaminyltransferase
262	302	73.43	12.69	YP_001128725.1	3-dehydroquinate dehydratase
263	323	73.4	17.67	YP_001129049.1	DNA replication intiation control protein YabA
264	172	73.38	14.49	YP_001128553.1	hypothetical protein SpyM50998
265	210	73.36	13.96	YP_001129340.1	recombination protein F
266	185	73.36	12.58	YP_001128440.1	GntR family transcriptional regulator
267	330	73.33	11.08	YP_001129076.1	phosphoesterase
268	246	73.21	12.79	YP_001128466.1	ribonuclease HII
269	332	73.1	15.29	YP_001129080.1	hypothetical protein SpyM51557
270	89	73	11.83	YP_001127817.1	NifU-like protein
271	140	72.81	12.38	YP_001128917.1	prolipoprotein diacylglyceryl transferase
272	337	72.52	16.04	YP_001129092.1	primosomal protein DnaI
273	339	72.33	18.12	YP_001129099.1	LemA family protein
274	132	72.3	14.58	YP_001128971.1	1,2-diacylglycerol 3-glucosyltransferase
275	218	72.18	12.45	YP_001127953.1	hypothetical protein SpyM50363
276	318	72.17	11.97	YP_001128989.1	formamidopyrimidine-DNA glycosylase
277	181	72.08	11.12	YP_001128413.1	16S rRNA m(2)G 1207 methyltransferase
278	261	71.96	21.45	YP_001128709.1	uracil permease
279	81	71.87	10.74	YP_001127831.1	hypothetical protein SpyM50240
280	160	71.85	13.70	YP_001128775.1	hypothetical protein SpyM51236
281	327	71.81	11.39	YP_001129072.1	segregation and condensation protein B
282	24	71.74	11.96	YP_001127944.1	acyl-carrier-protein S-malonyltransferase
283	153	71.56	13.73	YP_001128282.1	DNA polymerase III subunit delta
284	286	71.47	15.87	YP_001128674.1	mevalonate diphosphate decarboxylase
285	115	71.47	15.12	YP_001128193.1	geranyltranstransferase
286	303	71.41	12.47	YP_001128726.1	hypothetical protein SpyM51184
287	22	71.37	11.60	YP_001127941.1	3-oxoacyl-ACP synthase
288	284	71.25	16.18	YP_001128672.1	isopentenyl pyrophosphate isomerase
289	271	71.14	12.14	YP_001128625.1	NIF3 (NGG1p interacting factor 3) family protein
290	199	71.02	12.52	YP_001127923.1	universal stress protein
291	124	70.91	16.90	YP_001128800.1	zinc-binding protein AdcA precursor
292	289	70.83	12.19	YP_001128683.1	tRNA CCA-pyrophosphorylase
293	392	70.71	19.25	YP_001129297.1	arginine repressor
294	192	70.65	11.74	YP_001128670.1	3-hydroxy-3-methylglutaryl coenzyme A synthase
295	20	70.53	14.92	YP_001127939.1	enoyl-CoA hydratase
296	314	70.49	14.67	YP_001128363.1	4-alpha-glucanotransferase
297	275	70.41	15.27	YP_001128631.1	short chain dehydrogenase
298	178	70.34	13.85	YP_001128370.1	DNA polymerase III DnaE
299	94	70.22	16.73	YP_001127671.1	MarR family transcriptional regulator
300	195	70.17	14.78	YP_001128977.1	glucosyl transferase
301	348	69.97	12.08	YP_001127905.1	alanine racemase
302	209	69.78	17.06	YP_001129318.1	TetR family transcriptional regulator
303	365	69.71	13.49	YP_001127844.1	hypothetical protein SpyM50253
304	44	69.64	13.99	YP_001128415.1	lipoprotein
305	214	69.58	16.27	YP_001128023.1	penicillin-binding protein 1A
306	251	69.56	15.51	YP_001128478.1	hypothetical protein SpyM50921
307	328	69.54	12.55	YP_001129073.1	segregation and condensation protein A
308	232	69.49	15.93	YP_001128904.1	N-acetylmuramoyl-L-alanine amidase
309	213	69.4	17.17	YP_001129348.1	serine protease
310	46	69.33	16.73	YP_001127782.1	membrane protein OxaA 1 precursor
311	278	69.31	14.91	YP_001129006.1	uracil DNA glycosylase superfamily protein
312	131	69.16	21.67	YP_001128972.1	glycosyltransferase
313	45	69.05	14.79	YP_001127781.1	ribonuclease P
314	127	68.75	12.45	YP_001128322.1	3-phosphoshikimate 1-carboxyvinyltransferase
315	197	68.73	17.62	YP_001129084.1	OxaA-like protein precursor
316	272	68.71	12.74	YP_001128626.1	hypothetical protein SpyM51076
317	19	68.43	12.55	YP_001128047.1	hypothetical protein SpyM50461
318	233	68.41	16.57	YP_001128313.1	biotin--protein ligase
319	201	68.13	14.53	YP_001127898.1	sucrose-6-phosphate hydrolase
320	308	68.12	18.26	YP_001129055.1	polysaccharide biosynthesis protein
321	204	67.88	16.53	YP_001127876.1	signal peptidase I
322	296	67.65	14.64	YP_001128712.1	lipoprotein signal peptidase
323	85	67.65	17.62	YP_001127812.1	adaptor protein
324	273	67.61	16.28	YP_001128628.1	DNA replication protein DnaD
325	188	67.32	12.42	YP_001128501.1	cysteine desulfurase

326	110	67.17	13.24	YP_001128175.1	hypothetical protein SpyM50604
327	184	67.13	19.77	YP_001128472.1	sortase A
328	31	67.07	18.87	YP_001127958.1	permease
329	27	67.06	13.06	YP_001127947.1	acetyl-CoA carboxylase biotin carboxyl carrier protein subunit
330	8	67.06	19.49	YP_001128011.1	cell division protein
331	245	66.92	14.07	YP_001128397.1	bifunctional riboflavin kinase/FMN adenylyltransferase
332	17	66.74	19.07	YP_001128044.1	sensor histidine kinase
333	138	66.5	12.69	YP_001128928.1	cell division protein FtsY
334	389	66.02	16.91	YP_001129274.1	DNA-3-methyladenine glycosylase
335	186	65.89	14.38	YP_001128420.1	hypothetical protein SpyM50861
336	51	65.85	13.36	YP_001127635.1	phosphotyrosine protein phosphatase
337	141	65.71	18.02	YP_001128916.1	hypothetical protein SpyM51375
338	136	65.68	14.27	YP_001128952.1	chromosome partition protein
339	163	65.14	21.90	YP_001128771.1	F0F1 ATP synthase subunit A
340	252	65.04	14.62	YP_001128483.1	methyltransferase
341	203	64.95	12.80	YP_001127877.1	ribonuclease HIII
342	352	64.92	21.60	YP_001127899.1	PTS system sucrose-specific transporter subunit IIABC
343	32	64.84	17.48	YP_001127961.1	LytR family transcriptional regulator
344	300	64.78	19.20	YP_001128723.1	hypothetical protein SpyM51181
345	92	64.68	17.43	YP_001127824.1	oligopeptide transporter ATP-binding protein
346	384	64.57	16.66	YP_001129238.1	co-chaperonin GroES
347	355	64.46	21.04	YP_001127889.1	hypothetical protein SpyM50298
348	231	64.3	15.41	YP_001128907.1	phosphoglycerate mutase family protein
349	228	64.24	17.53	YP_001128871.1	metallo-beta-lactamase superfamily protein
350	277	63.91	16.90	YP_001128633.1	hypothetical protein SpyM51083
351	11	63.81	14.12	YP_001128025.1	hypothetical protein SpyM50438
352	187	63.51	15.85	YP_001128499.1	adenylate cyclase family protein
353	341	63.39	18.90	YP_001128504.1	peptidase
354	47	63.24	19.31	YP_001127783.1	ssDNA-binding protein
355	41	63.09	19.81	YP_001127976.1	copper-transporting ATPase
356	317	63.06	14.79	YP_001128988.1	dephospho-CoA kinase
357	224	63.03	18.73	YP_001127832.1	mechanosensitive ion channel protein
358	164	62.92	21.52	YP_001128770.1	F0F1 ATP synthase subunit B
359	93	62.58	18.95	YP_001127825.1	oligopeptide transporter ATP-binding protein
360	385	62.55	16.84	YP_001129263.1	acetyltransferase (GNAT) family protein
361	208	62.52	13.27	YP_001129234.1	RNA pseudouridylate synthase
362	217	62.46	13.93	YP_001128049.1	hypothetical protein SpyM50463
363	170	62.46	15.61	YP_001128753.1	ATP-dependent exonuclease subunit A
364	173	61.69	24.17	YP_001128552.1	hypothetical protein SpyM50997
365	53	61.31	16.50	YP_001127640.1	MATE family transporter
366	324	61.06	19.23	YP_001129050.1	DNA polymerase III subunit delta'
367	129	60.98	16.33	YP_001128324.1	cell envelope-related function transcriptional attenuator
368	80	60.85	14.50	YP_001127829.1	phosphomethylpyrimidine kinase
369	268	60.64	15.87	YP_001128568.1	hypothetical protein SpyM51016
370	116	60.48	22.35	YP_001128195.1	repressor protein
371	104	59.68	15.88	YP_001128158.1	phosphopantetheine adenylyltransferase
372	176	59.34	20.24	YP_001128548.1	acyl-ACP thioesterase
373	137	59.13	21.10	YP_001128930.1	haloacid dehalogenase-like hydrolase
374	282	59.09	17.93	YP_001128668.1	dihydrofolate reductase
375	165	59	22.86	YP_001128769.1	F0F1 ATP synthase subunit delta
376	379	58.98	21.46	YP_001129168.1	preprotein translocase subunit YajC
377	403	58.88	20.05	YP_001129335.1	hypothetical protein SpyM51822
378	52	58.52	17.99	YP_001127636.1	hypothetical protein SpyM50037
379	5	58.52	16.54	YP_001127614.1	PP-loop family protein
380	128	58.22	16.04	YP_001128323.1	shikimate kinase
381	338	57.91	17.47	YP_001129097.1	hypothetical protein SpyM51574
382	285	57.88	18.47	YP_001128673.1	phosphomevalonate kinase
383	4	57.83	18.77	YP_001127613.1	hypothetical protein SpyM50010
384	119	57.11	18.07	YP_001128889.1	hypothetical protein SpyM51348
385	212	56.87	16.88	YP_001129346.1	ABC transporter permease
386	111	56.57	18.19	YP_001128176.1	hypothetical protein SpyM50605
387	34	56.39	17.84	YP_001127965.1	ABC transporter
388	105	56.23	18.46	YP_001128161.1	hypothetical protein SpyM50590
389	312	56.13	17.98	YP_001129067.1	PAP2 superfamily protein
390	225	56.05	17.57	YP_001127820.1	D-alanyl-D-alanine carboxypeptidase
391	48	55.77	34.57	YP_001127790.1	N-acetylneuraminate lyase
392	361	54.69	17.98	YP_001127879.1	hypothetical protein SpyM50288
393	402	54.41	19.22	YP_001129334.1	protease
394	78	54.34	19.62	YP_001128433.1	GntR family transcriptional regulator
395	112	54.23	19.20	YP_001128178.1	RNA binding protein
396	99	54.16	20.99	YP_001127678.1	DNA binding protein
397	329	53.06	19.26	YP_001129074.1	site-specific tyrosine recombinase XerD
398	49	52.36	28.03	YP_001127791.1	ROK family protein
399	335	50.54	20.45	YP_001129089.1	hypothetical protein SpyM51566
400	287	50.5	23.81	YP_001127772.1	ABC transporter ATP-binding protein
401	142	48.99	24.37	YP_001128915.1	hypothetical protein SpyM51374
402	269	46.95	28.77	YP_001128182.1	Mut/NUDIX family protein
403	91	43.95	27.79	YP_001127823.1	oligopeptide transporter permease
404	288	42.63	22.63	YP_001127771.1	ABC transporter ATP-binding protein

Position	Sequence number	Genus Average identity	SD	Accession (S. pyogenes str. Manfredo)	Annotation
1	59	99.56	0.77	YP_001127646.1	30S ribosomal protein S19
2	72	98.58	1.32	YP_001127667.1	30S ribosomal protein S11
3	304	98.08	1.37	YP_001128728.1	50S ribosomal protein L20
5	62	97.89	1.83	YP_001127649.1	50S ribosomal protein L16
6	103	97.49	2.18	YP_001129139.1	50S ribosomal protein L13
7	63	97.37	2.47	YP_001127652.1	50S ribosomal protein L14
8	61	97.11	2.41	YP_001127648.1	30S ribosomal protein S3
10	298	96.6	2.57	YP_001128714.1	50S ribosomal protein L27
11	65	96.43	2.64	YP_001127654.1	50S ribosomal protein L5
12	60	96.23	4.74	YP_001127647.1	50S ribosomal protein L22
13	368	96.13	2.31	YP_001127805.1	translation elongation factor G
14	58	96.11	3.71	YP_001127645.1	50S ribosomal protein L2
16	147	95.77	2.11	YP_001128896.1	elongation factor Tu
17	76	95.77	2.14	YP_001127937.1	Chaperone protein DnaK
18	68	95.73	2.57	YP_001127659.1	30S ribosomal protein S5
21	2	95.24	1.96	YP_001127607.1	GTP-dependent nucleic acid-binding protein
22	370	95.19	3.29	YP_001127803.1	30S ribosomal protein S12
23	369	95.19	3.65	YP_001127804.1	30S ribosomal protein S7
24	57	95.11	4.89	YP_001127644.1	50S ribosomal protein L23
25	167	94.98	2.97	YP_001128766.1	H+-transporting ATP synthase beta chain
26	126	94.95	3.93	YP_001128787.1	phosphopyruvate hydratase
27	73	94.89	4.3	YP_001127668.1	DNA-directed RNA polymerase subunit alpha
28	97	94.82	3.29	YP_001127676.1	DNA-directed RNA polymerase subunit beta
29	66	94.31	3.6	YP_001127656.1	30S ribosomal protein S8
31	358	94.07	4.02	YP_001127886.1	30S ribosomal protein S6
32	372	93.96	3.13	YP_001127727.1	Adenylosuccinate synthetase
33	166	93.88	4.3	YP_001128768.1	H+-transporting ATP synthase alpha chain
34	98	93.88	3.84	YP_001127677.1	DNA-directed RNA polymerase subunit beta'
35	404	93.78	3.61	YP_001129342.1	inosine monophosphate dehydrogenase
36	43	93.72	3.59	YP_001127754.1	tRNA-guanine transglycosylase
38	253	93.58	3.44	YP_001128484.1	peptide chain release factor 1
39	319	93.44	4.72	YP_001129003.1	GTP binding proteins
40	64	93.37	3.73	YP_001127653.1	50S ribosomal protein L24
43	90	92.49	4.69	YP_001127818.1	FeS cluster assembly protein
45	69	91.98	4.4	YP_001127661.1	50S ribosomal protein L15
47	336	91.92	4.02	YP_001129091.1	GTP binding protein
49	237	91.17	5.33	YP_001128438.1	GMP synthetase
50	149	91.17	3.94	YP_001128270.1	recombination protein RecR
52	367	90.81	5.9	YP_001127806.1	glyceraldehyde 3-phosphate dehydrogenase
53	388	90.8	5.38	YP_001129272.1	recombination protein RecA
55	373	90.48	5.83	YP_001129255.1	translation elongation factor EF-Ts
57	174	89.91	5.53	YP_001128551.1	phospho-sugar mutase
58	230	89.86	5.47	YP_001128909.1	lysyl-tRNA synthetase
60	242	89.77	6.21	YP_001128404.1	phosphate ABC transporter (permease)
61	133	89.58	6.78	YP_001128956.1	two-component response regulator
62	320	89.4	6.33	YP_001129013.1	ribosome recycling factor
63	13	89.36	5.56	YP_001128036.1	guanylate kinase
64	67	89.33	5.52	YP_001127657.1	50S ribosomal protein L6
66	86	89.09	5.6	YP_001127814.1	ABC transporter (ATP-binding protein)
67	238	89.08	5.95	YP_001128442.1	signal recognition particle chain Ffh
69	179	88.88	7.07	YP_001128372.1	pyruvate kinase
70	364	88.82	5.25	YP_001127846.1	glutamine synthetase
72	130	88.69	8.96	YP_001128973.1	catabolite control protein A
73	346	88.65	6.6	YP_001127928.1	Glutamyl-tRNA Gln amidotransferase subunit A
74	169	88.64	7.74	YP_001128761.2	Phenylalanyl-tRNA synthetase alpha chain
77	333	88.42	6.75	YP_001129085.1	Transcription elongation factor greA
78	236	88.18	7.96	YP_001128474.1	L-Lactate Dehydrogenase
79	226	87.74	7.1	YP_001127674.1	tyrosyl-tRNA synthetase
80	280	87.55	6.58	YP_001128530.1	50S ribosomal protein L10
82	239	87.36	5.18	YP_001128441.1	putative DNA-binding protein
83	36	87.33	6.66	YP_001127969.1	transcription termination-antitermination factor nusA
84	394	87.09	6.98	YP_001129303.1	aspartyl-tRNA synthetase
85	206	87.04	8.6	YP_001127697.1	tRNA-dihydrouridine synthase family protein
86	326	86.96	6.55	YP_001129071.1	ribosomal large subunit pseudouridine synthase B (hypothetical)
87	223	86.96	6.31	YP_001127769.1	NAD(P)H-dependent glycerol-3-phosphate dehydrogenase
88	148	86.89	9.57	YP_001128895.1	triosephosphate isomerase
91	274	86.31	6.12	YP_001128629.1	adenine phosphoribosyltransferase
92	77	86.31	7.01	YP_001127938.1	Chaperone protein DnaJ
93	156	86.18	7.91	YP_001128308.1	uridine kinase
94	375	86.14	6.81	YP_001129164.1	prolyl-tRNA synthetase
96	222	85.91	5.75	YP_001127768.1	UDP-glucose pyrophosphorylase
97	50	85.87	5.59	YP_001127634.1	Holliday junction DNA helicase, subunit B
98	381	85.81	7.71	YP_001129175.1	GTP pyrophosphokinase (stringent response protein RelA)
99	40	85.79	8.24	YP_001127973.1	ribosome binding factor A
100	35	85.78	6.75	YP_001127967.1	tRNA (guanine-N(7)-)-methyltransferase

101	183	85.64	6.49	YP_001128473.1	DNA gyrase A subunit
104	371	85.45	8.27	YP_001127801.1	transcription repressor of purine operon PurR
105	158	85.34	6.55	YP_001128316.1	UDP-N-acetylglucosamine 1-carboxyvinyltransferase
108	154	85.27	8.49	YP_001128283.1	manganese-dependent superoxide dismutase
109	397	85.16	9.08	YP_001129321.1	replicative DNA helicase DnaC
110	113	85.13	8.05	YP_001128180.1	isoleucyl-tRNA synthetase
111	180	85.09	7.66	YP_001128374.1	glucosamine-fructose-6-phosphate aminotransferase
113	120	84.99	7.53	YP_001128873.1	cell division ATP-binding protein FtsE
114	18	84.85	9.33	YP_001128045.1	two-component response regulator
116	23	84.79	6.94	YP_001127943.1	putative trans-2-enoyl-ACP reductase II
119	315	84.64	7.25	YP_001128982.1	SsrA-binding protein
121	292	84.61	7.25	YP_001128694.1	tRNA (guanine-N1)-methyltransferase
123	395	84.44	7.91	YP_001129304.1	histidyl-tRNA synthetase
124	219	84.43	10.16	YP_001127954.1	mannose-specific PTS enzyme IID
125	71	84.4	7.98	YP_001127663.1	adenylate kinase
126	151	84.38	8.72	YP_001128275.1	ATP-dependent RNA helicase, DEAD-box family
127	334	84.29	6.74	YP_001129088.1	UDP-N-acetylmuramate-alanine ligase
128	30	84.27	9.13	YP_001127951.1	acetyl-coenzyme A carboxylase carboxyl transferase subunit alpha
129	12	84.25	7.67	YP_001128027.1	Hypothetical protein GBS222_0064
130	363	84.22	6.77	YP_001127851.1	putative DNA-binding/iron metalloprotein/AP endonuclease
131	139	84.22	9.37	YP_001128918.1	Hpr (ser) kinase/phosphatase
133	29	84.11	8.91	YP_001127950.1	acetyl-coenzyme A carboxylase carboxyl transferase subunit beta
134	374	84.1	9.2	YP_001129163.1	DNA polymerase III (alpha subunit)
135	276	84.09	8.53	YP_001128632.1	ribonuclease z
136	283	84.08	6.55	YP_001128669.1	thymidylate synthase
137	344	84.01	8.42	YP_001129114.1	probable nicotinate-nucleotide adenylyltransferase
139	309	83.94	8.28	YP_001129062.1	manganese-dependent inorganic pyrophosphatase
140	359	83.74	8.43	YP_001127882.1	thioredoxin
141	205	83.73	9.25	YP_001127698.1	Hsp33-like chaperonin
142	248	83.62	6.67	YP_001128654.1	orotate phosphoribosyltransferase
143	270	83.6	9.63	YP_001128572.1	mutator mutT protein
144	102	83.5	6.85	YP_001129144.1	tRNA/rRNA methyltransferase
145	260	83.4	8.46	YP_001128382.1	ATP-dependent DNA helicase
146	95	83.31	8.26	YP_001127672.1	(metal) ABC transporter (ATP-binding protein)
150	244	83.08	11.6	YP_001128398.1	transcriptional regulator Spx
153	123	82.79	8.96	YP_001128865.1	Hypothetical protein GBS222_0508
155	398	82.59	9.32	YP_001129322.1	50S ribosomal protein L9
156	25	82.46	8.84	YP_001127945.1	beta-ketoacyl-ACP reductase
157	311	82.33	9.47	YP_001129064.1	CBS domain-containing protein
158	347	82.3	10.64	YP_001127927.1	Glu-tRNA Gln amidotransferase subunit C
159	122	82.26	7.82	YP_001128866.1	Hypothetical protein GBS222_0507
161	175	82.01	9.33	YP_001128549.1	coproporphyrinogen III oxidase (HemN)
162	299	81.94	7.73	YP_001128718.1	hypothetical thiamine biosynthesis protein ThiI
163	177	81.69	10.26	YP_001128547.1	N-acetylglucosamine metabolism protein
164	26	81.63	10.15	YP_001127946.1	3-oxoacyl-acyl-carrier protein synthase
165	293	81.47	9.41	YP_001128695.1	Putative 16S rRNA processing protein RimM
166	295	81.29	8.36	YP_001128711.1	ribosomal large subunit pseudouridine synthase B (hypothetical)
167	390	81.15	10.37	YP_001129275.1	Holliday junction DNA helicase
168	354	81.13	18.45	YP_001127893.1	late competence protein required for DNA binding and uptake comEB
169	227	80.87	9.67	YP_001128264.1	dihydroorotate dehydrogenase A
170	42	80.84	8.48	YP_001127749.1	DNA polymerase I
171	171	80.79	8.66	YP_001128561.1	ABC transporter (ATP-binding protein)
172	109	80.67	11.18	YP_001128173.1	cell division protein FtsA
174	255	80.51	11.97	YP_001128497.1	inorganic polyphosphate/ATP-NAD kinase
176	259	80.4	10.28	YP_001128520.1	UDP-N-acetylenolpyruvoylglucosamine reductase
177	313	80.38	8.44	YP_001128747.1	Hypothetical protein GBS222_1175
178	279	80.34	10.98	YP_001128533.1	dipeptidase
179	378	80.25	10.06	YP_001129167.1	undecaprenyl pyrophosphate synthetase
180	87	80.18	9.17	YP_001127815.1	Hypothetical protein GBS222_0291
181	1	80.1	13.94	YP_001127604.1	Chromosomal replication initiator protein DnaA
182	342	80.07	11.75	YP_001129111.1	Hypothetical protein GBS222_1384
183	306	80.02	11.43	YP_001128731.1	cytidine monophosphate kinase
184	380	79.95	10.26	YP_001129174.1	D-tyrosyl-tRNA(Tyr) deacylase
185	207	79.88	9.33	YP_001129160.1	polypeptide deformylase
186	96	79.79	10.32	YP_001127673.1	(metal) ABC transporter (permease)
188	150	79.59	8.58	YP_001128271.1	D-alanine-D-alanine ligase
189	190	79.57	11.63	YP_001128503.1	redox-sensing transcriptional repressor Rex
190	33	79.55	9.49	YP_001127964.1	ABC transporter (ATP-binding protein)
193	307	79.31	10.88	YP_001128735.1	tripeptidase
194	198	79.2	8.71	YP_001129100.1	Ribosomal RNA small subunit methyltransferase
195	215	79.18	9.68	YP_001128024.1	recombination protein U (RecU)
196	101	79.1	9.52	YP_001129146.1	Hypothetical protein GBS222_0353
197	193	79.09	10.84	YP_001128469.1	hemolysin III
199	353	79.01	13.7	YP_001127895.1	Hypothetical protein GBS222_1420
200	400	78.91	11.59	YP_001129329.1	putative ABC transporter (permease)
202	79	78.61	8.27	YP_001127828.1	tRNA pseudouridine synthase A
203	125	78.59	12.79	YP_001128795.1	flavodoxin

205	331	78.38	8.98	YP_001129078.1	glutamate racemase
207	155	78.2	9.54	YP_001128291.1	methyltransferase
209	88	78.13	8.31	YP_001127816.1	aminotransferase, putative cysteine desulfurase
210	38	78.08	12.1	YP_001127971.1	putative ribosomal protein
212	360	77.98	11.68	YP_001127880.1	DNA mismatch repair protein MutS
213	75	77.9	11.78	YP_001127936.1	heat shock protein GrpE
215	114	77.87	11.7	YP_001128191.1	exodeoxyribonuclease VII large chain
216	134	77.83	13.26	YP_001128955.1	two-component sensor histidine kinase
219	393	77.72	12.78	YP_001129302.1	Hypothetical protein GBS222_1711
220	168	77.65	14.79	YP_001128765.1	H+-transporting ATP synthase epsilon chain
221	39	77.63	9.26	YP_001127972.1	initiation factor 2
222	74	77.49	13.08	YP_001127935.1	transcription repressor of class I heat-shock (HrcA)
224	9	77.33	10.96	YP_001128013.1	phospho-N-acetylmuramoyl-pentapeptide transferase
225	152	77.29	12.41	YP_001128279.1	acyltransferase family protein
226	316	77.22	11.95	YP_001128983.1	exoribonuclease R
228	135	76.99	11.03	YP_001128953.1	ribonuclease III
230	350	76.82	11.08	YP_001127902.1	mannose-6-phosphate isomerase
231	121	76.67	9.55	YP_001128868.1	aspartate aminotransferase
233	21	76.53	14.43	YP_001127940.1	transcriptional regulator (MarR family)
234	229	76.52	13.74	YP_001128923.1	Hypothetical protein GBS222_0602
235	161	76.49	9.14	YP_001128774.1	DNA ligase
236	162	76.24	20.85	YP_001128773.1	putative lipid kinase
237	301	76.1	9.31	YP_001128724.1	chorismate synthase
238	107	75.98	9.61	YP_001128170.1	UDP-N-acetylmuramoylalanine--D-glutamate ligase
239	321	75.97	10.18	YP_001129017.1	DNA translocase
240	249	75.93	10.13	YP_001128655.1	orotidine 5-phosphate decarboxylase
241	377	75.62	13.17	YP_001129166.1	phosphatidate cytidylyltransferase (CDP-diglyceride synthase)
243	146	75.01	13.22	YP_001128898.1	cell cycle protein FtsW
244	157	74.88	12.56	YP_001128311.1	GAF domain-containing protein
245	291	74.68	13.86	YP_001128693.1	thioredoxin reductase
246	15	74.62	12.67	YP_001128040.1	RNA-binding protein Sun
247	382	74.61	12.42	YP_001129180.1	Hypothetical protein GBS222_1630
248	16	74.56	14.93	YP_001128041.1	serine/threonine protein phosphatase Stp1
249	401	74.41	11.01	YP_001129331.1	ABC transporter (ATP-binding protein)
253	235	74.18	11.92	YP_001128412.1	pantothenate kinase
254	322	74.12	11.89	YP_001129026.1	ADP-ribose pyrophosphatase
255	3	74.08	11.86	YP_001127608.1	peptidyl-tRNA hydrolase
256	216	74.06	9.95	YP_001128029.2	autoinducer-2 /production protein (LuxS)
257	343	73.79	13.71	YP_001129113.1	HD domain-containing protein
258	100	73.64	13.95	YP_001127686.1	Hypothetical protein GBS222_0316
259	6	73.57	14.68	YP_001127991.1	N-acetylglucosamine-6-phosphate deacetylase
260	221	73.45	13.04	YP_001127963.1	cell-cycle regulation histidine triad (HIT) protein
261	108	73.45	10.62	YP_001128171.1	undecaprenyl-PP-MurNAc-pentapeptide-UDPGlcNAc GlcNAc transferase
262	302	73.43	12.69	YP_001128725.1	3-dehydroquinate dehydratase
264	172	73.38	14.49	YP_001128553.1	Hypothetical protein GBS222_0756
265	210	73.36	13.96	YP_001129340.1	recombination protein RecF
266	185	73.36	12.58	YP_001128440.1	transcriptional regulator (GntR family)
267	330	73.33	11.08	YP_001129076.1	Hypothetical protein GBS222_1330
270	89	73	11.83	YP_001127817.1	NifU protein
271	140	72.81	12.38	YP_001128917.1	prolipoprotein diacylglycerol transferase
272	337	72.52	16.04	YP_001129092.1	primosome component (helicase loader) DnaI
274	132	72.3	14.58	YP_001128971.1	glucosyl transferase
276	318	72.17	11.97	YP_001128989.1	formamidopyrimidine-DNA glycosylase
277	181	72.08	11.12	YP_001128413.1	E. coli 16S RNA m2G1207 methyltransferase
281	327	71.81	11.39	YP_001129072.1	segregation and condensation protein B
282	24	71.74	11.96	YP_001127944.1	malonyl CoA-acyl carrier protein transacylase
283	153	71.56	13.73	YP_001128282.1	DNA polymerase III subunit delta
285	115	71.47	15.12	YP_001128193.1	geranyltranstransferase (farnesyl diphosphate synthase)
286	303	71.41	12.47	YP_001128726.1	Hypothetical protein GBS222_1133
287	22	71.37	11.6	YP_001127941.1	beta-ketoacyl-ACP synthase III
288	284	71.25	16.18	YP_001128672.1	Isopentenyl diphosphate isomerase
290	199	71.02	12.52	YP_001127923.1	hypothetical protein GBS222_1403
292	289	70.83	12.19	YP_001128683.1	poly(A) polymerase family protein
295	20	70.53	14.92	YP_001127939.1	enoyl-CoA isomerase
297	275	70.41	15.27	YP_001128631.1	oxidoreductase
298	178	70.34	13.85	YP_001128370.1	DNA polymerase III (alpha subunit)
300	195	70.17	14.78	YP_001128977.1	putative sugar transferase
301	348	69.97	12.08	YP_001127905.1	alanine racemase
304	44	69.64	13.99	YP_001128415.1	putative ABC transporter (binding protein)
305	214	69.58	16.27	YP_001128023.1	penicillin-binding protein 1A
306	251	69.56	15.51	YP_001128478.1	Hypothetical protein GBS222_0896
307	328	69.54	12.55	YP_001129073.1	segregation and condensation protein A
310	46	69.33	16.73	YP_001127782.1	membrane protein oxaA, putative
315	197	68.73	17.62	YP_001129084.1	OxaA-like protein precursor
317	19	68.43	12.55	YP_001128047.1	general stress protein GSP13
318	233	68.41	16.57	YP_001128313.1	transcriptional repressor of the biotin operon
320	308	68.12	18.26	YP_001129055.1	Hypothetical protein GBS222_1143

321	204	67.88	16.53	YP_001127876.1	signal peptidase 1
324	273	67.61	16.28	YP_001128628.1	DNA replication protein DnaD, putative
325	188	67.32	12.42	YP_001128501.1	iron-sulfur cofactor synthesis protein or cysteine desulfurase
326	110	67.17	13.24	YP_001128175.1	Hypothetical protein GBS222_0458
328	31	67.07	18.87	YP_001127958.1	Xanthine/uracil permease family protein
339	163	65.14	21.9	YP_001128771.1	H+-transporting ATP synthase a chain
341	203	64.95	12.8	YP_001127877.1	Ribonuclease HII
342	352	64.92	21.6	YP_001127899.1	sucrose-specific PTS enzyme IIABC
345	92	64.68	17.43	YP_001127824.1	oligopeptide ABC transporter (ATP-binding protein)
349	228	64.24	17.53	YP_001128871.1	metallo-beta-lactamase family protein
351	11	63.81	14.12	YP_001128025.1	Hypothetical protein GBS222_0062
355	41	63.09	19.81	YP_001127976.1	copper-transporting ATPase (CopA)
356	317	63.06	14.79	YP_001128988.1	dephosphocoenzyme A kinase
359	93	62.58	18.95	YP_001127825.1	putative oligopeptide ABC transporter, ATP-binding protein
362	217	62.46	13.93	YP_001128049.1	Hypothetical protein GBS222_0092
363	170	62.46	15.61	YP_001128753.1	ATP-dependent exonuclease, subunit A
364	173	61.69	24.17	YP_001128552.1	Hypothetical protein GBS222_0757
374	282	59.09	17.93	YP_001128668.1	dihydrofolate reductase
377	403	58.88	20.05	YP_001129335.1	zinc dependent peptidase
381	338	57.91	17.47	YP_001129097.1	Hypothetical protein GBS222_1356
385	212	56.87	16.88	YP_001129346.1	unknown transmembrane protein
390	225	56.05	17.57	YP_001127820.1	serine-type D-Ala-D-Ala carboxypeptidase (penicilin binding protein)
391	48	55.77	34.57	YP_001127790.1	N-acetylneuraminate lyase, putative
395	112	54.23	19.2	YP_001128178.1	S4 domain-containing protein
398	49	52.36	28.03	YP_001127791.1	glucose kinase and to XylR transcriptional regulator
404	288	42.63	22.63	YP_001127771.1	ABC transporter, ATP-binding/permease protein

Position	Sequence number	Genus Average identity	SD	Accession (S. pyogenes str. Manfredo)	Annotation
4	54	97.93	1.81	YP_001127641.1	30S ribosomal protein S10
9	396	97.1	1.8	YP_001129319.1	30S ribosomal protein S4
15	55	96.01	2.75	YP_001127642.1	50S ribosomal protein L3
19	366	95.45	2.79	YP_001127843.1	phosphoglycerate kinase
20	106	95.45	2.89	YP_001128168.1	putative GTP-binding elongation factor
30	56	94.21	4.97	YP_001127643.1	50S ribosomal protein L4
37	211	93.66	3.68	YP_001129345.1	ABC transporter (ATP-binding protein)
41	325	93.11	4.58	YP_001129053.1	ATP-dependent CLP protease proteolytic subunit
42	305	92.89	6.45	YP_001128730.1	translation initiation factor IF-3
44	399	92.14	3.32	YP_001129325.1	tRNA (5-methylaminomethyl-2-thiouridylate)-methyltransferase
46	383	91.94	3.87	YP_001129237.1	chaperonin GroEL
48	262	91.58	5.19	YP_001128642.1	ribosomal protein S1-like DNA-binding protein
51	28	91.01	3.84	YP_001127948.1	beta-hydroxyacyl-ACP dehydratase
54	387	90.61	6.5	YP_001129271.1	transcriptional regulator Spx
56	265	90.01	5.04	YP_001128647.1	DNA topoisomerase IV (subunit B)
59	14	89.83	6.38	YP_001128037.1	Hypothetical protein GBS222_0074
65	159	89.29	5.7	YP_001128320.1	methionine aminopeptidase
68	84	88.96	6.15	YP_001127837.1	fructose-bisphosphate aldolase class-II
71	234	88.82	5.67	YP_001128532.1	putative tRNA modification GTPase TrmE
75	144	88.6	7.06	YP_001128912.1	U32 family peptidase
76	220	88.53	5.92	YP_001127956.1	PTS system, mannose-specific IIAB components
81	386	87.48	6.44	YP_001129266.1	anaerobic (class III) ribonucleotide reductase large subunit chain
89	345	86.61	7.38	YP_001129116.1	GTP-binding protein YqeH
90	263	86.41	7.35	YP_001128644.1	branched-chain-amino-acid aminotransferase
95	241	86.02	7.76	YP_001128406.1	phosphate ABC transporter (ATP-binding protein)
102	356	85.54	11.11	YP_001127890.1	Putative transport protein
103	196	85.47	7.13	YP_001129044.1	3-exo-deoxyribonuclease
106	349	85.33	7.43	YP_001127903.1	preprotein translocase SecA subunit
107	281	85.32	6.41	YP_001128665.1	hypothetical GTP-binding protein
112	37	85.03	8.04	YP_001127970.1	Hypothetical protein GBS222_0134
115	357	84.81	9.25	YP_001127887.1	single strand binding protein
117	70	84.71	10.34	YP_001127662.1	preprotein translocase SecY
118	250	84.69	6.78	YP_001128430.1	formate-tetrahydrofolate ligase
120	182	84.63	9.81	YP_001128475.1	NADH Oxidase
122	258	84.59	8.5	YP_001128518.1	spermidine/putrescine ABC transporter (permease)
132	254	84.19	9.69	YP_001128485.1	thymidine kinase
138	191	84.01	7.72	YP_001128657.1	transcriptional regulator (LysR family)
147	257	83.28	9	YP_001128517.1	spermidine/putrescine ABC transporter (permease)
148	82	83.27	7.6	YP_001127833.1	trigger factor (prolyl isomerase)
149	294	83.26	10.18	YP_001128710.1	pyrimidine biosynthetic operon repressor
151	264	82.84	8.9	YP_001128645.1	topoisomerase IV subunit A
152	83	82.84	8.1	YP_001127774.1	deoxyuridine 5-triphosphate nucleotidhydrolase (dUTPase)
154	266	82.74	9.4	YP_001128564.1	metallo-beta-lactamase family protein
160	240	82.24	11.62	YP_001128407.1	phosphate uptake regulatory protein
173	310	80.57	10.34	YP_001129063.1	pyruvate-formate lyase activating enzyme
175	7	80.45	9.29	YP_001127995.1	glycyl-tRNA synthetase (beta subunit)
187	391	79.74	10.11	YP_001129277.1	DNA mismatch repair MutL
191	247	79.46	10.79	YP_001128649.1	dihydroorotase
192	290	79.4	8.24	YP_001128689.1	Fructose-1-phosphate kinase
198	143	79.03	12.44	YP_001128913.1	U32 family peptidase
201	243	78.73	11.61	YP_001128402.1	Phosphate ABC transporter, phosphate-binding protein
204	267	78.48	9.61	YP_001128566.1	ABC transporter (permease)
206	200	78.2	9.18	YP_001127921.1	L-asparaginase
208	117	78.14	11.59	YP_001128196.1	DNA repair and recombination protein RecN
211	256	78.05	13.32	YP_001128516.1	spermidine/putrescine ABC transporter (binding protein)
214	362	77.88	12.19	YP_001127858.1	Hypothetical protein GBS222_1473
217	351	77.78	9.51	YP_001127901.1	fructokinase
218	194	77.73	9.85	YP_001128349.1	glutamine ABC transporter permease and substrate binding protein
223	189	77.4	10.21	YP_001128502.1	Hypothetical protein GBS222_0922
227	340	77.19	12.78	YP_001129105.1	ABC transporter (permease)
229	202	76.94	14.08	YP_001127897.1	transcriptional regulator (LacI family)
232	10	76.67	13.06	YP_001128015.1	amino acid ABC transporter (permease)
242	297	75.31	12.69	YP_001128713.1	transcriptional regulator (LysR/MarR family)
250	376	74.38	11.7	YP_001129165.1	M50A family peptidase
251	145	74.36	10.86	YP_001128899.1	phosphoenolpyruvate carboxylase
252	118	74.28	11.77	YP_001128198.1	DegV family protein
263	323	73.4	17.67	YP_001129049.1	DNA replication intiation control protein YabA
268	246	73.21	12.79	YP_001128466.1	ribonuclease HII
269	332	73.1	15.29	YP_001129080.1	Hypothetical protein GBS222_1336
273	339	72.33	18.12	YP_001129099.1	LemA family protein
275	218	72.18	12.45	YP_001127953.1	Hypothetical protein GBS222_0114
278	261	71.96	21.45	YP_001128709.1	uracil permease
279	81	71.87	10.74	YP_001127831.1	Hypothetical protein GBS222_0254
280	160	71.85	13.7	YP_001128775.1	Hypothetical protein GBS222_0720

284	286	71.47	15.87	YP_001128674.1	mevalonate diphosphate decarboxylase
289	271	71.14	12.14	YP_001128625.1	NIF3 family protein
291	124	70.91	16.9	YP_001128800.1	metal ABC transporter (binding protein)
293	392	70.71	19.25	YP_001129297.1	arginine repressor ArgR
294	192	70.65	11.74	YP_001128670.1	3-hydroxy-3-methylglutaryl-coenzyme A synthase (HMG-CoA synthase)
296	314	70.49	14.67	YP_001128363.1	4-alpha-glucanotransferase (amylomaltase)
299	94	70.22	16.73	YP_001127671.1	transcriptional regulator
302	209	69.78	17.06	YP_001129318.1	transcriptional regulator (TetR/AcrR family)
303	365	69.71	13.49	YP_001127844.1	Hypothetical protein GBS222_1486
308	232	69.49	15.93	YP_001128904.1	bacteriophage endolysin
309	213	69.4	17.17	YP_001129348.1	serine protease
311	278	69.31	14.91	YP_001129006.1	Hypothetical protein GBS222_1047
312	131	69.16	21.67	YP_001128972.1	hexosyltransferase
313	45	69.05	14.79	YP_001127781.1	ribonuclease P protein component
314	127	68.75	12.45	YP_001128322.1	5-enolpyruvylshikimate-3-phosphate synthase
316	272	68.71	12.74	YP_001128626.1	Hypothetical protein GBS222_1032
319	201	68.13	14.53	YP_001127898.1	sucrose-6-phosphate hydrolase
322	296	67.65	14.64	YP_001128712.1	prolipoprotein signal peptidase
323	85	67.65	17.62	YP_001127812.1	competence negative regulator mecA
327	184	67.13	19.77	YP_001128472.1	sortase family protein
329	27	67.06	13.06	YP_001127947.1	biotin carboxyl carrier protein
330	8	67.06	19.49	YP_001128011.1	cell division protein FtsL
331	245	66.92	14.07	YP_001128397.1	macrolide-efflux protein
332	17	66.74	19.07	YP_001128044.1	two-component sensor histidine kinase
333	138	66.5	12.69	YP_001128928.1	signal recognition particle and to cell division protein FtsY
334	389	66.02	16.91	YP_001129274.1	3-methyl-adenine DNA glycosylase I
335	186	65.89	14.38	YP_001128420.1	Hypothetical protein GBS222_0891
336	51	65.85	13.36	YP_001127635.1	protein-tyrosine phosphatase
337	141	65.71	18.02	YP_001128916.1	Hypothetical protein GBS222_0611
338	136	65.68	14.27	YP_001128952.1	chromosome segregation SMC protein
340	252	65.04	14.62	YP_001128483.1	HemK family modification methylase
343	32	64.84	17.48	YP_001127961.1	transcription regulator, hypothetical
344	300	64.78	19.2	YP_001128723.1	Hypothetical protein GBS222_1129
346	384	64.57	16.66	YP_001129238.1	chaperonin GroES
347	355	64.46	21.04	YP_001127889.1	Hypothetical protein GBS222_1431
348	231	64.3	15.41	YP_001128907.1	putative phosphoglycerate mutase
350	277	63.91	16.9	YP_001128633.1	Hypothetical protein GBS222_1039
352	187	63.51	15.85	YP_001128499.1	hypothetical protein GBS222_0919
353	341	63.39	18.9	YP_001128504.1	glutamine amidotransferase class I
354	47	63.24	19.31	YP_001127783.1	R3H domain-containing protein
357	224	63.03	18.73	YP_001127832.1	Hypothetical protein GBS222_0255
358	164	62.92	21.52	YP_001128770.1	H+-transporting ATP synthase b chain
360	385	62.55	16.84	YP_001129263.1	acetyltransferase
361	208	62.52	13.27	YP_001129234.1	ribosomal large subunit pseudouridine synthase A
365	53	61.31	16.5	YP_001127640.1	MATE efflux family protein
366	324	61.06	19.23	YP_001129050.1	DNA polymerase III (delta subunit)
367	129	60.98	16.33	YP_001128324.1	membrane bound transcriptional regulator
368	80	60.85	14.5	YP_001127829.1	phosphomethylpyrimidine kinase
369	268	60.64	15.87	YP_001128568.1	hypothetical protein GBS222_1018
370	116	60.48	22.35	YP_001128195.1	transcriptional regulator, arginine repressor, putative
371	104	59.68	15.88	YP_001128158.1	phosphopantetheine adenyltransferase
372	176	59.34	20.24	YP_001128548.1	thioesterase
373	137	59.13	21.1	YP_001128930.1	Cof-like hydrolase family protein
375	165	59	22.86	YP_001128769.1	H+-transporting ATP synthase delta chain
376	379	58.98	21.46	YP_001129168.1	preprotein translocase subunit YajC
378	52	58.52	17.99	YP_001127636.1	Hypothetical protein GBS222_0202
379	5	58.52	16.54	YP_001127614.1	tRNA(Ile)-lysidine synthase
380	128	58.22	16.04	YP_001128323.1	shikimate kinase
382	285	57.88	18.47	YP_001128673.1	phosphomevalonate kinase
383	4	57.83	18.77	YP_001127613.1	weakly beta-lactamase
384	119	57.11	18.07	YP_001128889.1	Hypothetical protein GBS222_0490
386	111	56.57	18.19	YP_001128176.1	Hypothetical protein GBS222_0459
387	34	56.39	17.84	YP_001127965.1	ABC transporter (permease)
388	105	56.23	18.46	YP_001128161.1	Hypothetical protein GBS222_0435
389	312	56.13	17.98	YP_001129067.1	PAP2 family protein
392	361	54.69	17.98	YP_001127879.1	Hypothetical protein GBS222_1441
393	402	54.41	19.22	YP_001129334.1	zinc dependent peptidase
394	78	54.34	19.62	YP_001128433.1	GntR family transcriptional regulator
396	99	54.16	20.99	YP_001127678.1	Hypothetical protein GBS222_0311
397	329	53.06	19.26	YP_001129074.1	integrase
399	335	50.54	20.45	YP_001129089.1	Hypothetical protein GBS222_1348
400	287	50.5	23.81	YP_001127772.1	ABC transporter, ATP-binding/permease protein
401	142	48.99	24.37	YP_001128915.1	Hypothetical protein GBS222_0612
402	269	46.95	28.77	YP_001128182.1	NUDIX family hydrolase
403	91	43.95	27.79	YP_001127823.1	oligopeptide ABC transporter (permease)

Gene ID	Genus Average Identity	SD	Reference Accession	Annotation
8	97.46	1.7	YP_007968185.1	30S ribosomal protein S9
33	96.95	2.1	YP_007968954.1	50S ribosomal protein L11
1	95.71	2.1	YP_007967888.1	glycyl-tRNA synthetase (alpha subunit)
14	94.43	3.7	YP_007968324.1	ribosomal protein L19
45	92.38	3.9	YP_007969161.1	metallo-beta-lactamase family protein
4	92.22	5.2	YP_007968011.1	ribose-phosphate pyrophosphokinase
38	91.86	4.1	YP_007969003.1	uracil phosphoribosyltransferase
9	91.28	4.4	YP_007968234.1	valyl-tRNA synthetase
41	90.85	4.8	YP_007969060.1	Hypothetical protein GBS222_1374
15	90.56	4.6	YP_007968327.1	DNA gyrase, subunit B
21	89.73	4.8	YP_007968478.1	S-adenosylmethionine synthetase
31	89.4	6.9	YP_007968914.1	Glutamine ABC transporter ATP-binding protein
46	89.04	6.1	YP_007969179.1	16 rRNA (adenine-N6,N6-)-dimethyltransferase
24	88.56	5.7	YP_007968644.1	phosphoglucomutase
50	88.08	5.8	YP_007969294.1	recombination factor protein RarA
2	86.13	7.2	YP_007967906.1	ATP-dependent RNA helicase
22	86.08	6.5	YP_007968606.1	DNA topoisomerase I
6	85.88	7.6	YP_007968117.1	Hypothetical protein GBS222_0281
12	85.62	8.7	YP_007968297.1	cation-transporting P-ATPase
10	85.54	6.2	YP_007968273.1	ATP-dependent protease ClpE
35	84.96	7.0	YP_007968982.1	methionyl-tRNA synthetase
32	84.59	7.6	YP_007968938.1	putative metalloprotease
3	82.66	9.1	YP_007967945.1	ribosomal subunit interface protein
49	81.63	9.2	YP_007969286.1	ribosomal protein L11 methyltransferase
40	80.47	10.7	YP_007969055.1	ABC transporter (ATP-binding protein)
27	79.98	9.6	YP_007968750.1	hypothetical fibronectin-binding protein
7	79.31	9.9	YP_007968176.1	serine acetyltransferase
18	78.81	12.5	YP_007968389.1	permease, putative
34	78.55	9.3	YP_007968969.1	UDP-N-acetylglucosamine pyrophosphorylase
20	78.41	11.8	YP_007968456.1	group B oligopeptidase PepB
43	78.09	14.3	YP_007969075.1	HAD superfamily hydrolase
11	77.94	10.3	YP_007968278.1	bifunctional methylenetetrahydrofolate dehydrogenase / methenyltetrahydrofolate cyclohydrolase (FolD)
48	77.11	13.9	YP_007969224.1	transctiptional regulator CtsR
42	76.5	12.5	YP_007969069.1	iojap-related protein
16	75.86	15.5	YP_007968328.1	negative regulator of FtsZ ring formation protein EzrA
39	75.84	8.9	YP_007969005.1	rRNA methylase
17	75.6	11.8	YP_007968336.1	23S rRNA (uracil-5-)-methyltransferase RumA
47	75.56	11.3	YP_007969183.1	TatD family hydrolase
30	73.18	23.4	YP_007968903.1	preprotein translocase secA
28	72.18	12.8	YP_007968771.1	tRNA isopentenylpyrophosphate transferase
26	71.19	11.7	YP_007968720.1	putative glycerol-3-phosphate acyltransferase PlsY
36	70.88	14.5	YP_007968988.1	hypothetical protein GBS222_1298
37	70.8	12.2	YP_007968997.1	thymidylate kinase
13	70.56	13.4	YP_007968319.1	DHH family protein
19	70.11	14.3	YP_007968451.1	N-acetylglucosamine-6-phosphate isomerase
5	67.86	11.8	YP_007968013.1	DNA repair protein RecO
23	66.25	20.3	YP_007968630.1	cardiolipin synthetase
51	62.62	16.9	YP_007969410.1	Partitioning protein, ParB family
29	61.95	18.3	YP_007968866.1	Hypothetical protein GBS222_1156
25	60.18	18.9	YP_007968686.1	folyl-polyglutamate synthetase
44	54.89	21.9	YP_007969090.1	shikimate 5-dehydrogenase
