# Supplementary material for: Global genomic similarity and core genome sequence diversity of the Streptococcus genus as a toolkit to identify closely related bacterial species in complex environments
Source: PeerJ. 2019 Jan 14;6:e6233. doi: 10.7717/peerj.6233 (PMC6336011; doi:10.7717/peerj.6233)
Supplement: Supplemental Information 4 — GSS calculations jupyter notebook. [file peerj-07-6233-s004.pdf]

Supplementary methods

Compute GSS

The following code was used to compute the GSS for each pair of proteomes. It was run on python v2.7 and used BLAST+ v2.2.28 and GNU Awk v4.0.1.

Load libraries:

In [ ]:
```
import sys
import string
import itertools
import os
```

Set paths:

In [ ]:
```
path_bin = '/usr/bin/' # path to the python program
path_db = './' # path to the fasta files
path_out = './' # path to save the output
```

Load the input file streptococcus_proteomes.txt, it contains a list of the names of the proteome files to compare:

In [ ]:
```
handle = open('streptococcus_proteomes.txt','r')
filenames = handle.readlines()
handle.close()
```

Make a list of the file names and generate all combinations of pairwise comparisons:

In [ ]:
```
proteomes = []
for name in filenames:
    proteomes.append(string.strip(name))

proteome_per = itertools.permutations(proteomes,2)
```

Build the BLAST databases:

In [ ]:
```
os.system("while read filename; do makeblastdb -in " + path_db + "$fi
lename -dbtype 'prot'; done < "+ infilename)
```

Run the BLASTp searches for the pairwise comparisons:

In [ ]:
```
for pair in proteome_per:
    os.system( path_bin + 'blastp -query '+ path_db + pair[0]+' -db '
+ path_db + pair[1]+" -outfmt '6 qseqid sseqid pident length mismatch
 gapopen qstart qend sstart send evalue bitscore qcovs' -evalue 1e-6
 -soft_masking "+'"true"'+' -use_sw_tback -max_target_seqs 10 > ' + p
ath_out + pair[0]+'-'+pair[1]+'.comp')
```

Run a BLASTp seach for each proteome against itself:

In [ ]:
```
for prot in proteomes:
    os.system( path_bin + 'blastp -query ' + path_db +prot+' -db ' +
path_db +prot+' -outfmt 6 -evalue 1e-6 -soft_masking "true" -use_sw_t
back -max_target_seqs 10 > ' + path_out +prot+'.self.tblout')
```

Filter the BLAST output alignments by query coverage, all hits with a coverage < 60% are discarded:

In [ ]:
```
proteomes_per = itertools.permutations(proteomes,2)
for pair in proteomes_per:
    os.system("awk '"+'{if($NF >= 60) print $1"\t"$2"\t"$3"\t"$4"\t
"$5"\t"$6"\t"$7"\t"$8"\t"$9"\t"$10"\t"$11"\t"$12}'+"' "+ path_out +pa
ir[0]+'-'+pair[1]+'.comp > '+ path_out +pair[0]+'-'+pair[1]+'.comp.tb
lout')
```

Define the function to compare the BLAST output tables and retrieve the reciprocal best hits (or bidirectional best hits):

In [ ]:
```
def bbh(first,second,outfilename):
    handle = open(first,'r')
    tabl1 = handle.readlines()
    handle.close()
    tabl1uniq = []
    tabl1dict = {}
    for line in tabl1:
        line = string.strip(line)
        line_sep = string.split(line,'\t')
        if line_sep[0] not in tabl1uniq:
            tabl1dict[line_sep[0]] = [line_sep[0],line_sep[1],line_se
p[11]]
        tabl1uniq.append(line_sep[0])
    handle = open(second,'r')
    tabl2 = handle.readlines()
    handle.close()
    tabl2list = []
    tabl2uniq = []
    for line in tabl2:
        line = string.strip(line)
        line_sep = string.split(line,'\t')
        if line_sep[0] not in tabl2uniq:
            nline = [line_sep[0],line_sep[1],line_sep[11]]
            tabl2list. append(nline)
        tabl2uniq.append(line_sep[0])
    outfile = open(outfilename,'w')
    for line in tabl2list:
        if line[1] in tabl1dict.keys():
            if tabl1dict[line[1]][1] == line[0]:
                outfile.write( tabl1dict[line[1]][0]+'\t'+tabl1dict[l
ine[1]][1]+'\t'+tabl1dict[line[1]][2]+'\n')
    outfile.close()
```

Get the reciprocal best hits:

In [ ]:
```
proteomes_comb = itertools.combinations(proteomes,2)
for pair in proteomes_comb:
        bbh(path_out + pair[0]+'-'+pair[1]+'.comp.tblout', path_out +
 pair[1]+'-'+pair[0]+'.comp.tblout', path_out + pair[0]+'-'+pair[1]+
'.comp.tblout-'+pair[1]+'-'+pair[0]+'.comp.tblout.bbhs')
```

Extract scores from the reciprocal best hit files:

In [ ]:
```
proteomes_comb = itertools.combinations(proteomes,2)
for pair in proteomes_comb:
    os.system("awk '{print $3}' "+ path_out +pair[0]+"-"+pair[1]+".co
mp.tblout-"+ pair[1]+"-"+pair[0]+".comp.tblout.bbhs > "+ path_out +pa
ir[0]+"-"+pair[1]+".comp.tblout-"+ pair[1]+"-"+pair[0]+".comp.tblout.
bbhs.num")
```

Save the ortholog pairs in a dictionary for further comparisons:

In [ ]:
```
ortholog_files = {}
proteomes_comb = itertools.combinations(proteomes,2)
for pair in proteomes_comb:
    handle = open(path_out + pair[0]+"-"+pair[1]+".comp.tblout-"+ pair[1]+"-"+pair[0]+".comp.tblout.bbhs",'r')
    ortholog_files[pair[0]+"-"+pair[1]+".comp.tblout-"+ pair[1]+"-"+pair[0]+".comp.tblout.bbhs"] = handle.read()
    handle.close()
```

Get the sums of the bitscores for each ortholog file:

In [ ]:
```
rbh_sum = {}
proteomes_comb = itertools.combinations(proteomes,2)
for pair in proteomes_comb:
    handle = open(path_out +pair[0]+"-"+pair[1]+".comp.tblout-"+ pair[1]+"-"+pair[0]+".comp.tblout.bbhs.num",'r')
    rbh_sum[pair[0]+"-"+pair[1]+".comp.tblout-"+ pair[1]+"-"+pair[0]+".comp.tblout.bbhs.num"] = handle.readlines()
    handle.close()
    numbers = 0
    for line in rbh_sum[pair[0]+"-"+pair[1]+".comp.tblout-"+ pair[1]+"-"+pair[0]+".comp.tblout.bbhs.num"]:
        numbers += float(string.strip(line))
    rbh_sum[pair[0]+"-"+pair[1]+".comp.tblout-"+ pair[1]+"-"+pair[0]+".comp.tblout.bbhs.num"] = numbers
```

Format the self-blasts output to keep only the accession number of each protein and the bitscore of the alignment agains itself.

In [ ]:
```
self_num = {}
for name in proteomes:
    os.system("awk '{if($1 == $2) print $1, $12}' "+ path_out + name +".self.tblout > "+ path_out + name +".self.tblout.num")
    handle = open(path_out + name +".self.tblout.num")
    self_num[name] = handle.readlines()
    handle.close()
```

Compute the genome similarity score and save an upper similarity matrix in the file streptococcus_proteomes.txt.gss.upper and a list of scores in the file streptococcus_proteomes.txt.gss.txt:

In [ ]:
```
outfile = open (infilename+'.gss.txt','w')
outtable = open(infilename+'.gss.upper.csv','w')
outtable.write('names,'+string.join(proteomes,','))
print
print ('Genome similarity scores:')
outfile.write('Genome similarity scores:\n')
```

```
proteomes_comb = itertools.combinations(proteomes,2)
last = 'null'
           # row zero in the upper matrix
index = 0
           # column zero in the upper matrix
for pair in proteomes_comb:
    comparison = rbh_sum[pair[0]+"-"+pair[1]+".comp.tblout-"+ pair[1]
+"-"+pair[0]+".comp.tblout.bbhs.num"]     # comparison = ortholog bit
score sum of the current pair
    sumself_first = 0

    for line in self_num[pair[0]]:
           # sumself_first = ortholog self bitscore of first partner

        sep_line = string.split(string.strip(line),' ')
        if sep_line[0] in ortholog_files[pair[0]+"-"+pair[1]+".comp.t
blout-"+ pair[1]+"-"+pair[0]+".comp.tblout.bbhs"]: # only takes into
 account self bitscores of genes present in ortholog list
            sumself_first += float(sep_line[1])
    sumself_second = 0
    for line in self_num[pair[1]]:
           # sumself_second = ortholog self bitscore of second partne
r
        sep_line = string.split(string.strip(line),' ')
        if sep_line[0] in ortholog_files[pair[0]+"-"+pair[1]+".comp.t
blout-"+ pair[1]+"-"+pair[0]+".comp.tblout.bbhs"]: # only takes into
 account self bitscores of genes present in ortholog list
            sumself_second += float(sep_line[1])
    if sumself_first == 0: # When there are no shared bbhs the calcul
ation below gives an error
        gss = 0.0
    else:
        gss = ((comparison/sumself_first) + (comparison/sumself_secon
d))/2      # compute Genome Similarity Score
    print pair[0]+' vs '+pair[1]+': '+str(gss)
    outfile.write(pair[0]+' vs '+pair[1]+': '+str(gss)+'\n')
    if last != pair[0]:                                        # check
 if the current cell belongs to the current row (i.e. the current par
tner belongs to the current pair), if not:
        outtable.write('\n'+pair[0]+','+(index*','))         #      m
ove to the next row and add empty cells
        outtable.write('1')                                    #      t
his cell belongs to the diagonal (the GSS of every proteome agains it
self equals 1)
        index += 1                                             #      f
or every new row in the upper matrix, there is one less column of sco
res, prepare an additional empty cell to the next row
    last = pair[0]                                             # save t
he first partner of the current pair
    outtable.write(','+str(gss))                               # add a
 new cell and write the current GSS
outfile.close()
outtable.write('\n'+proteomes[-1]+','+(index*','))
outtable.write('1')
outtable.close()
```

Build the Neighbor Joining tree

Code for computing the 1-GSS matrix and Neighbor Joining tree, it was run on R v3.3.1 with the package APE v3.5

Load APE and the GSS upper matrix:

In [ ]:
```
library(ape)
gss.raw <- read.csv("streptococcus_proteomes.txt.gss.upper",row.names
=1)
```

Build a symmetric similarity matrix and calculate the 1-GSS distance matrix:

In [ ]:
```
gss.final <- gss.raw
gss.trans <- t(as.matrix(gss.raw))
gss.final[lower.tri(gss.final)] <- gss.trans[lower.tri(gss.trans)]
gss.final <- 1-gss.final
```

Compute the Neighbor Joining tree nad save it in newik format:

In [ ]:
```
gss.tree <- nj(as.dist(gss.final))
write.tree(gss.tree, file="streptococcus_proteomes.txt.gss.tree")
```
