# Supplementary material for: Global genomic similarity and core genome sequence diversity of the Streptococcus genus as a toolkit to identify closely related bacterial species in complex environments
Source: PeerJ. 2019 Jan 14;6:e6233. doi: 10.7717/peerj.6233 (PMC6336011; doi:10.7717/peerj.6233)
Supplement: Supplemental Information 6 — Additionally, each streptococci species core genome and orthologous genes shared between strains. [file peerj-07-6233-s006.pdf]

Core genome

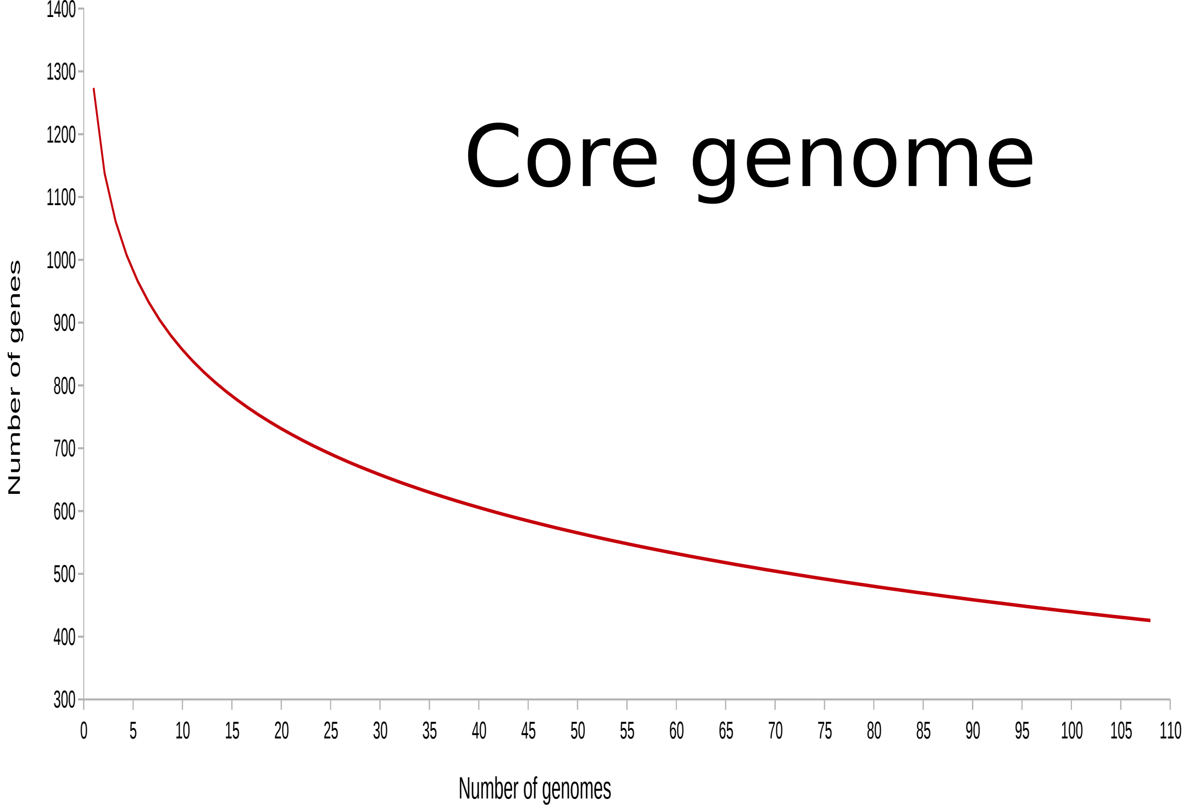

Pangenome

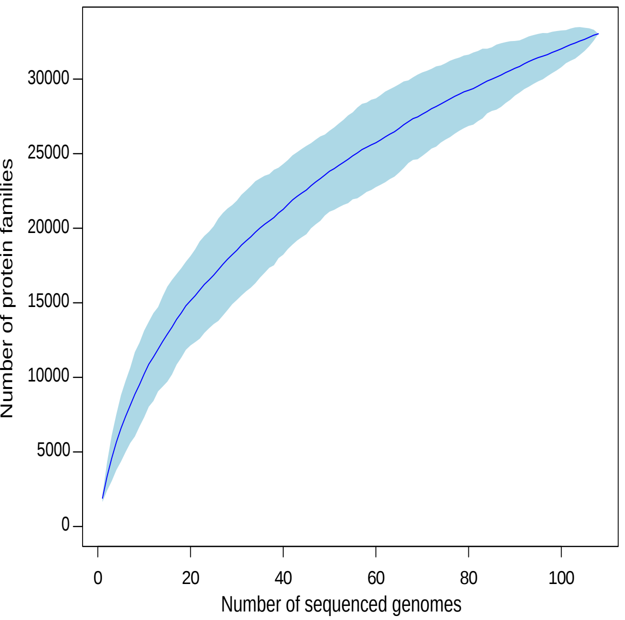

Species core genome

Number of genomes	Species	Number of core proteins	Average number of proteins
3	*S. gallolyticus*	1964	2266 +/- 56
3	*S. salivarius*	1646	1982 +/- 43
4	*S. mutans*	1627	1914 +/- 36
4	*S. dysgalactiae*	1491	2061 +/- 140
4	*S. suis*	1438	1962 +/- 101
9	*S. thermophilus*	1370	1973 +/- 207
16	*S. pneumoniae*	1334	1999 +/- 104
6	*S. thermophilus*	1314	1915 +/- 137
24	*S. pneumoniae*	1310	2035 +/- 165
19	*S. pyogenes*	1213	1808 +/- 111
