# Supplementary figures and images for: Global genomic similarity and core genome sequence diversity of the Streptococcus genus as a toolkit to identify closely related bacterial species in complex environments
Source: PeerJ. 2019 Jan 14;6:e6233. doi: 10.7717/peerj.6233 (PMC6336011; doi:10.7717/peerj.6233)

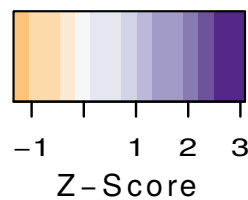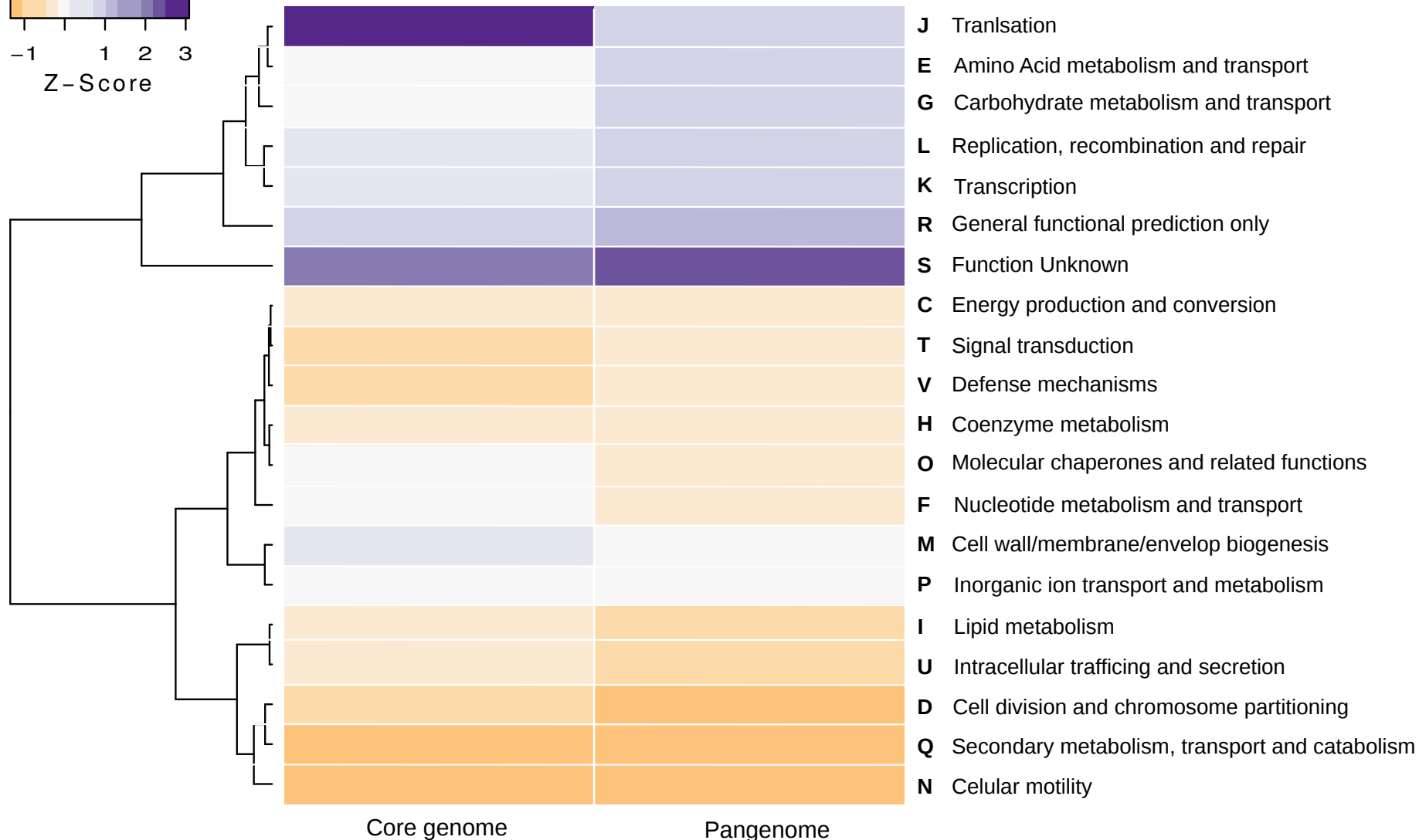

Supplement: Supplemental Information 7 [file peerj-07-6233-s007.pdf]

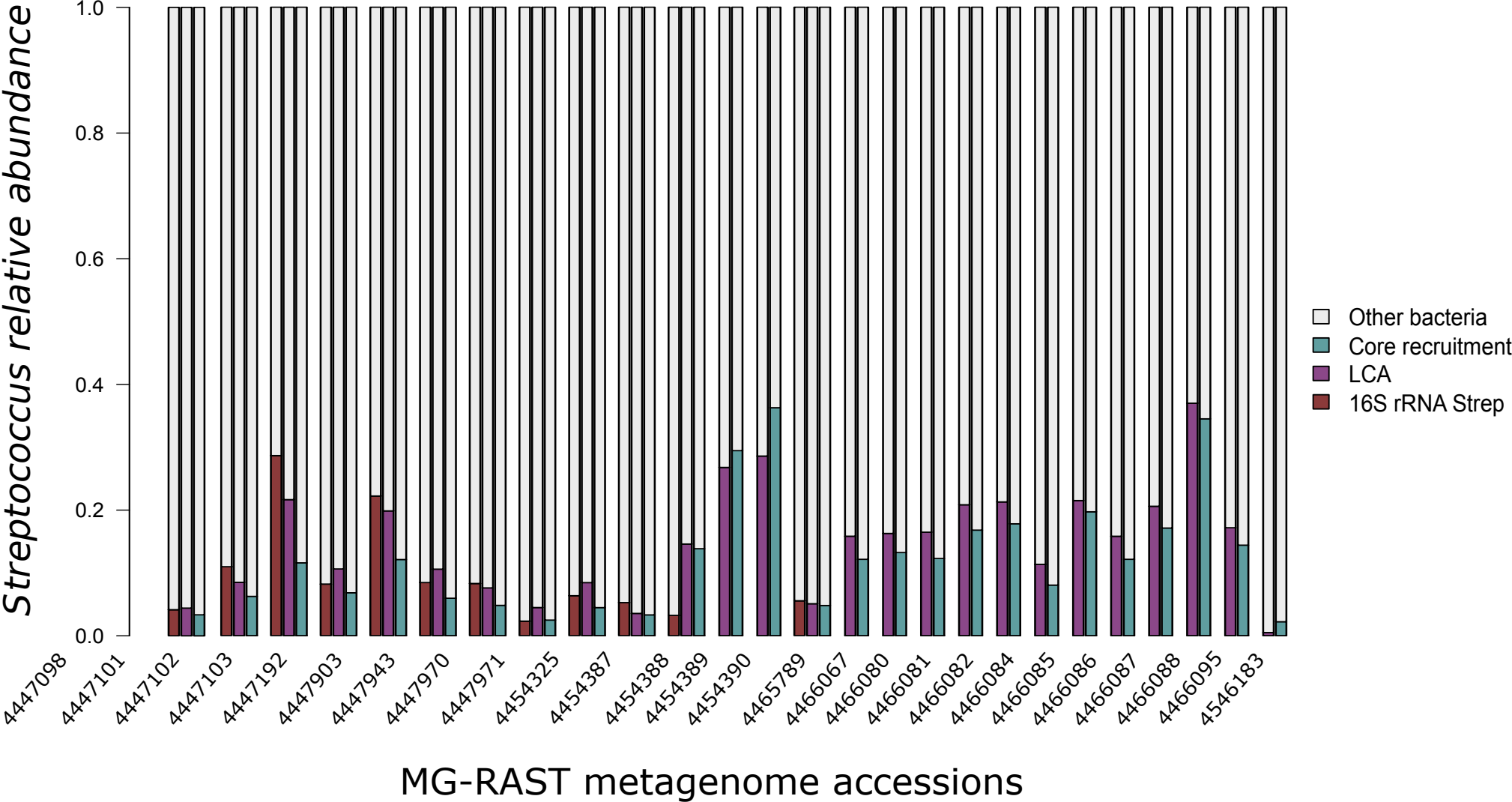

Supplement: Supplemental Information 8 — Calculated by core genome fragment recruitment, the lowest common ancestor (LCA), and 16S rRNA gene abundances. [file peerj-07-6233-s008.pdf]

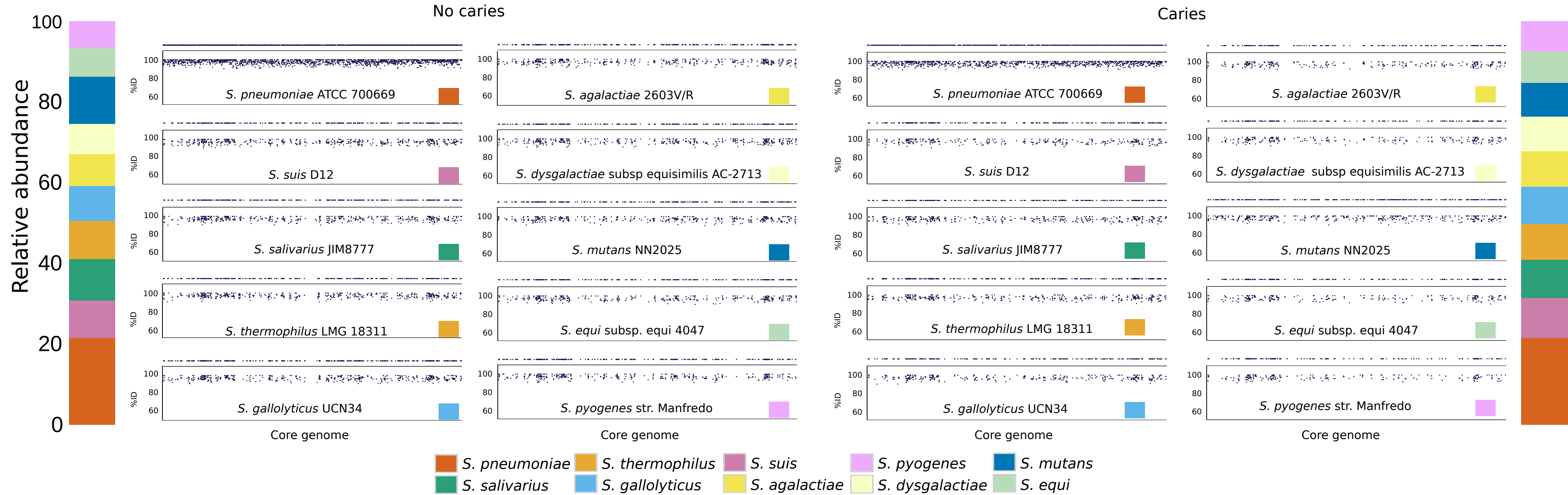

Supplement: Supplemental Information 9 — (A) Histogram showing the species gene relative abundance in metagenomic reads from a caries patient. (B) Metagenomic reads from a patient with caries, and C) Metagenomic reads from a healthy individual (caries-free) were aligned against the core genomes of 10 different species of Streptococci. (D) Histogram representing the species gene relative abundance in metagenomic reads from a healthy individual. Species-specific profiles can be generated by this method. S. mutans is depleted in the healthy individual while abundant metagenomic reads are identified in caries patient. [file peerj-07-6233-s009.pdf]

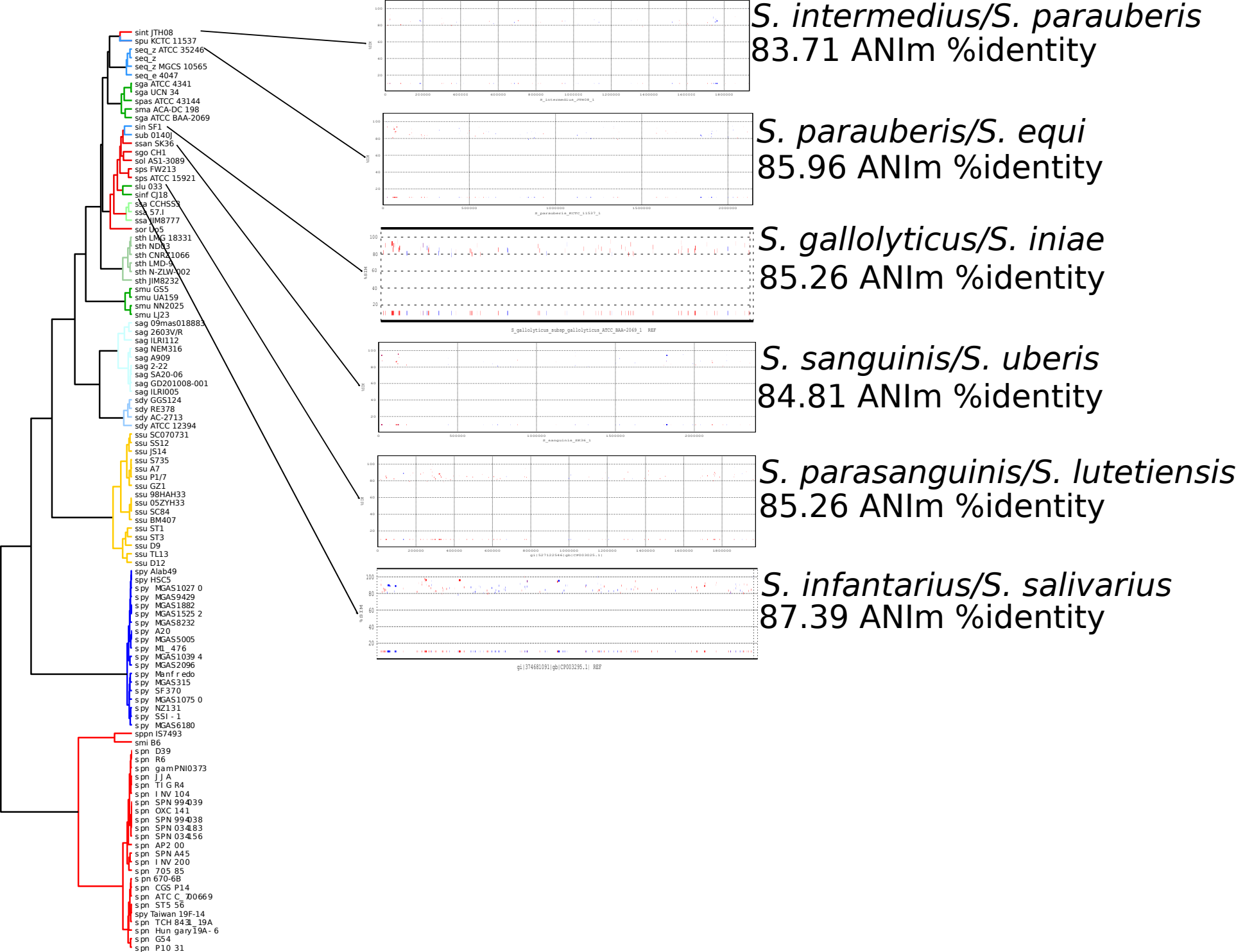

Supplement: Supplemental Information 10 [file peerj-07-6233-s010.pdf]

**A****Streptococcus groups**

- Pyogenic
- Suis
- Salivarius
- Mutans
- Mitis

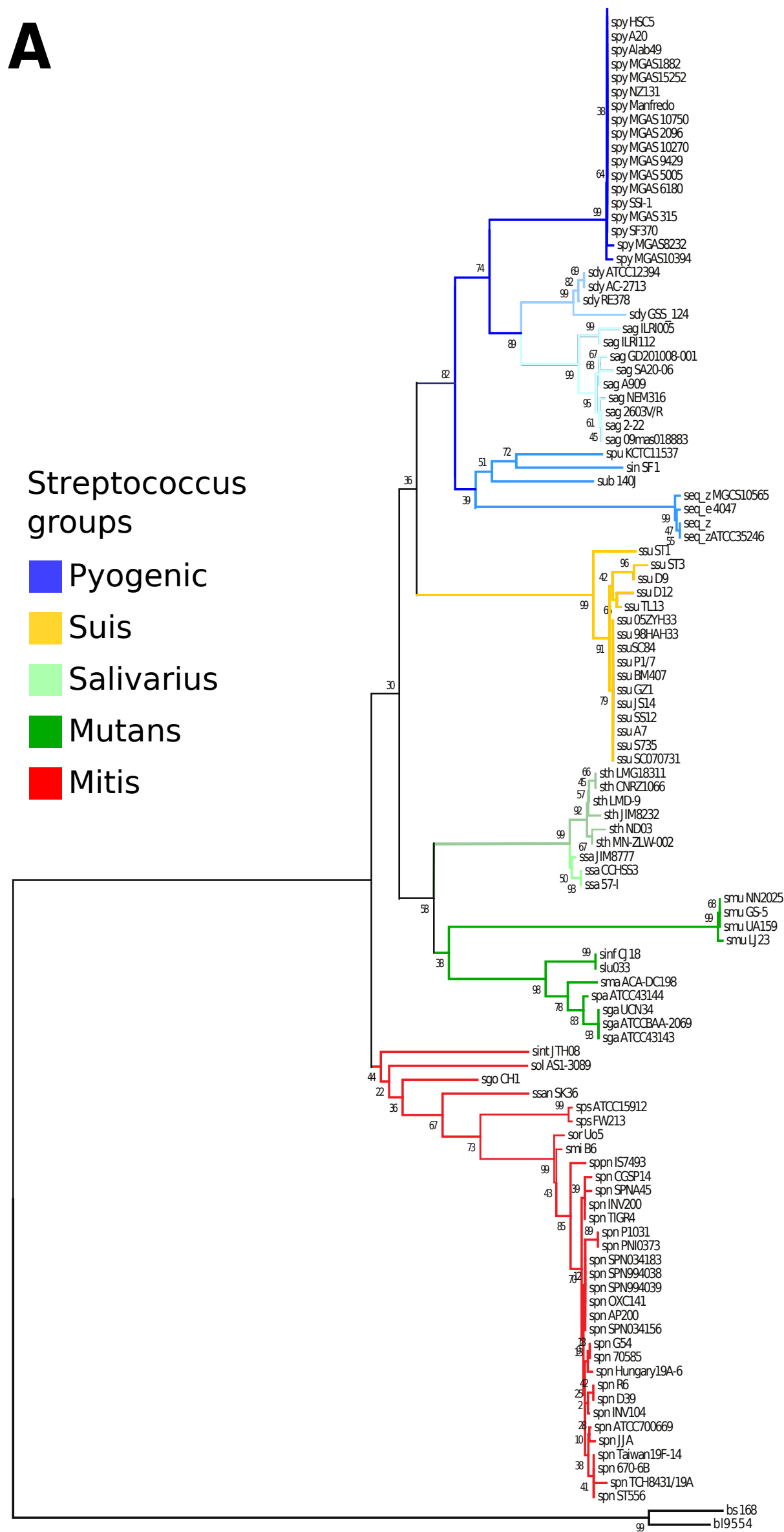**B**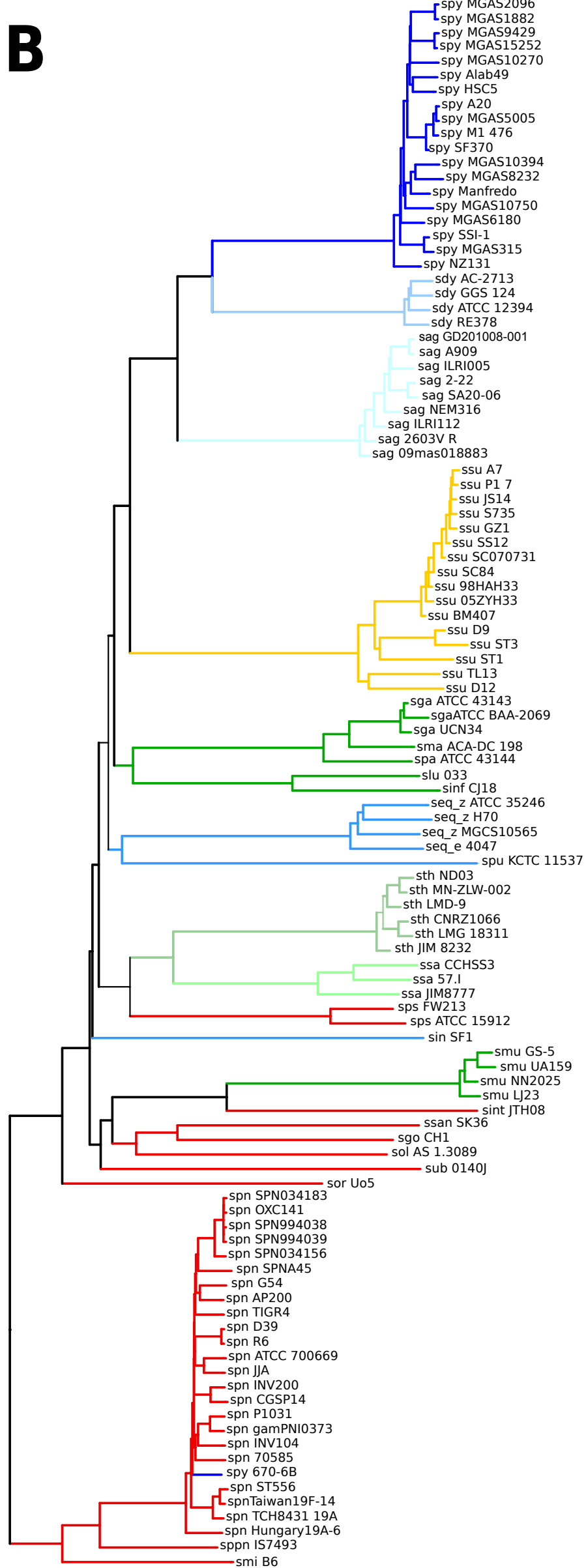**C**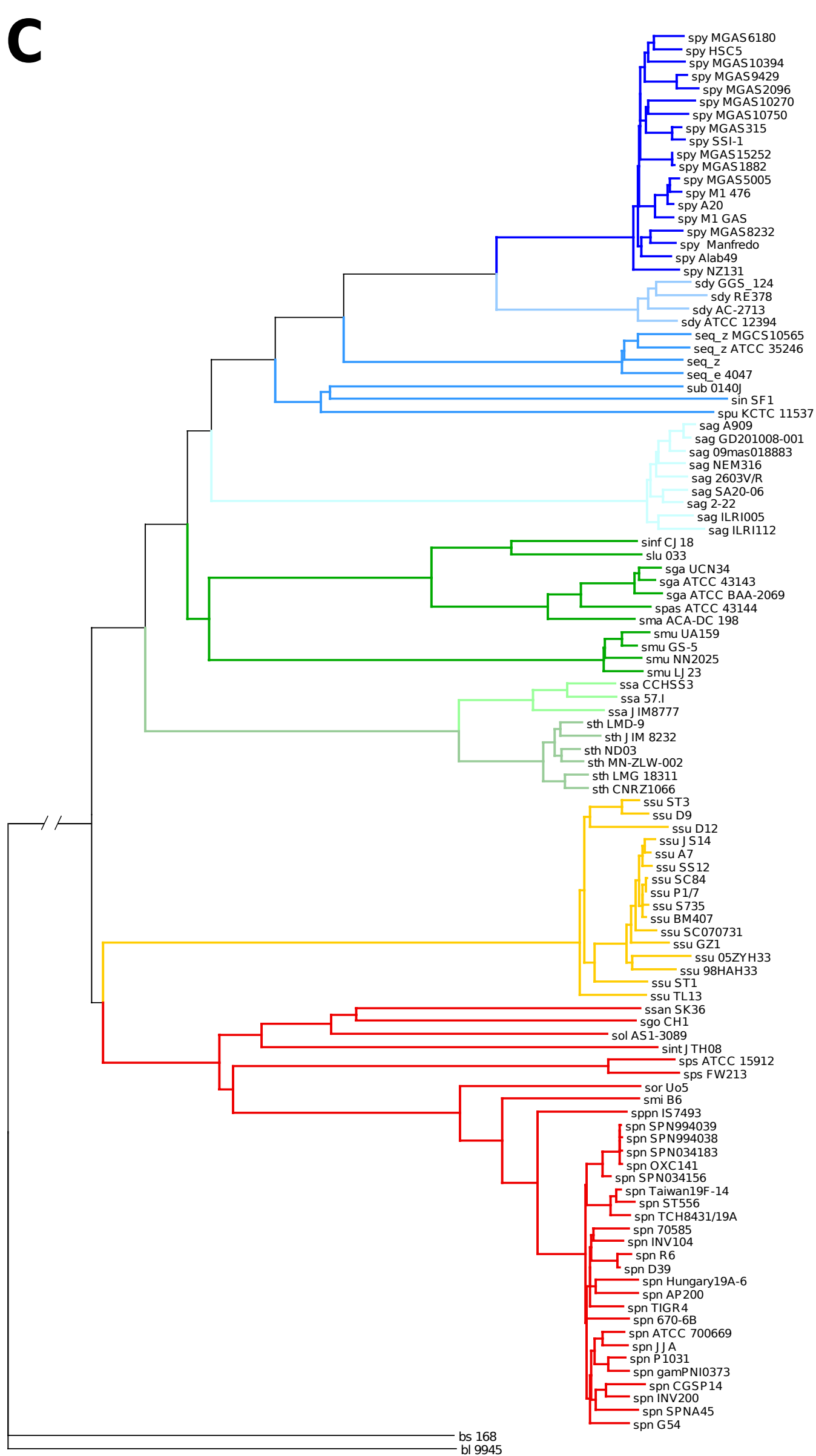

Supplement: Supplemental Information 11 [file peerj-07-6233-s011.pdf]

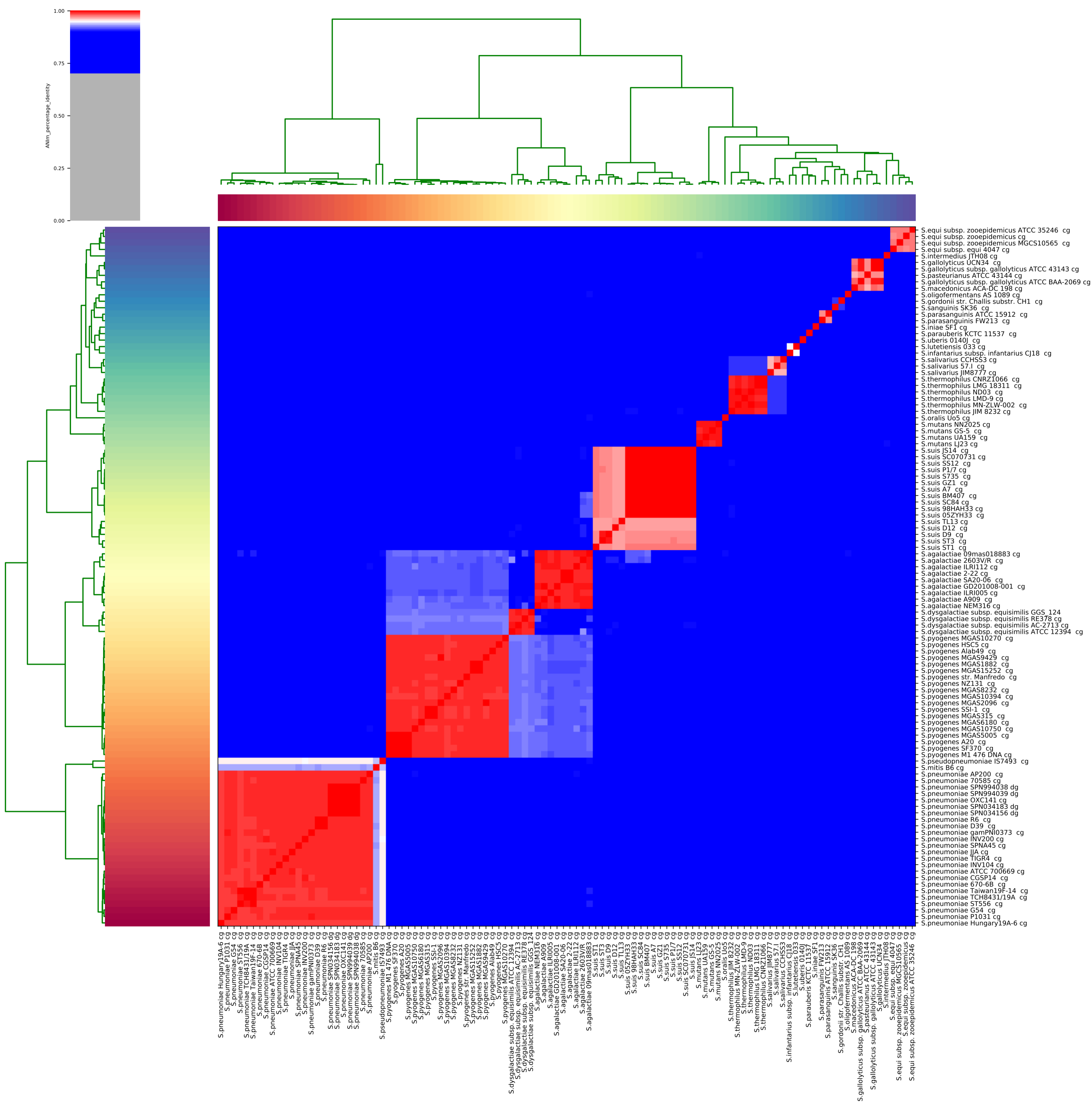

Supplement: Supplemental Information 12 [file peerj-07-6233-s012.pdf]
